# Equilibrium Swelling of Biocompatible Thermo-Responsive Copolymer Gels

**DOI:** 10.3390/gels7020040

**Published:** 2021-04-01

**Authors:** Aleksey D. Drozdov

**Affiliations:** Department of Materials and Production, Aalborg University, Fibigerstraede 16, 9220 Aalborg, Denmark; aleksey@m-tech.aau.dk

**Keywords:** thermo-responsive gel, biocompatible gel, copolymer gel, swelling, volume phase transition

## Abstract

Biomedical applications of thermo-responsive (TR) hydrogels require these materials to be biocompatible, non-cytotoxic, and non-immunogenic. Due to serious concerns regarding potential toxicity of poly(*N*-isopropylacrylamide) (PNIPAm), design of alternative homo- and copolymer gels with controllable swelling properties has recently become a hot topic. This study focuses on equilibrium swelling of five potential candidates to replace PNIPAm in biomedical and biotechnological applications: poly(*N*-vinylcaprolactam), poly(vinyl methyl ether), poly(N,N-dimethyl amino ethyl methacrylate), and two families of poly(2-oxazoline)s, and poly(oligo(ethylene glycol) methacrylates). To evaluate their water uptake properties and to compare them with those of substituted acrylamide gels, a unified model is developed for equilibrium swelling of TR copolymer gels with various types of swelling diagrams. Depending on the strength of hydrophobic interactions (high, intermediate, and low), the (co)polymers under consideration are split into three groups that reveal different responses at and above the volume phase transition temperature.

## 1. Introduction

Thermo-responsive (TR) gels form to a special group of stimuli-sensitive hydrogels whose equilibrium water uptake is strongly affected by temperature *T*. TR gels of the low critical solution temperature (LCST) type (also known as gels with negative temperature-sensitivity or thermophobic gels [1]) swell noticeably at temperatures below their volume phase transition temperature (VPTT) $T_{c}$ and collapse above VPTT [2]. Equilibrium and transient swelling of TR macroscopic and microgels gels has recently attracted substantial attention due to a wide spectrum of their potential biomedical applications [3] as smart materials for (i) controlled delivery and release of drugs and genes [4,5], (ii) adhesive dressings for wound healing [6], and (iii) scaffolds for tissue regeneration [7].

Poly(*N*-isopropylacrylamide) (PNIPAm) is the most extensively studied member of the family of temperature-sensitive polymers. PNIPAm gels exhibit abrupt volume phase transitions at temperature close to $T_{c}=32$ °C (this temperature is relatively insensitive to variations of preparation conditions, molar fractions of monomers and cross-linkers, as well as pH and molar fraction of salts under physiological conditions), demonstrate a strong reduction in the equilibrium degree of swelling *Q* at $T_{c}$, and show good mechanical properties both below and above VPTT [8]. As VPTT of PNIPAm homopolymer gels is lower than the physiological temperature, $T_{c}$ can be easily modulated by (i) copolymerization with (neutral or ionic) monomers whose hydrophilicity exceeds that of NIPAm monomers (copolymer gels) [9,10] and (ii) incorporation of hydrophilic polymer chains into pre-gel solutions (gels with inter-penetrating networks) [11].

The main drawback of substituted acrylamides (this group involves *N*-isopropylacrylamide (NIPAm), *N*-isopropylmethacrylamide (NIPMAm), *N*-ethylacrylamide (NEAm), N,N-diethylacrylamide (DEAm), *N*-methyl acrylamide (NMAm), N,N-dimethylacrylamide (DEAm), N,n-propylacrylamide (NNPAm), and *N*-cyclopropylacrylamide (NCPAm) [12,13]) is that these polymers can release potentially toxic small amine molecules, which leads to unwanted side effects during long-term applications. Analysis of cytotoxicity of PNIPAm has revealed that this material is toxic for all cell types [14,15,16], which is ascribed to the presence of unreacted monomers or toxic moieties arising under hydrolysis of chains. Although conflicting conclusions have been reported about biocompatibility and cytotoxicity of substituted acrylamide gels [17,18], serious concerns exist regarding their potential toxicity.

Two approaches have been proposed to avoid this shortcoming: (i) to reduce cytotoxicity of PNIPAm gels by chemical modification [19], and (ii) to search for alternative temperature-sensitive gels with controllable VPTT [20].

Five groups of biocompatible thermo-responsive polymers have become a focus of attention in the past decade: (i) poly(*N*-vinylcaprolactam) (PVCL), (ii) poly(vinyl methyl ether) (PVME), (iii) poly(N,N-dimethylaminoethyl methacrylate) (PDMAEMA), and two large families of (iv) poly(2-oxazoline)s (POx), and (v) poly(oligo(ethylene glycol) (meth)acrylates) (POEGMA).

The objective of this study is threefold: (i) to report a unified model for equilibrium swelling of covalently cross-linked TR homo- and copolymer gels and to demonstrate its ability to describe experimental data on the above hydrogels, (ii) to compare material parameters determined by matching observations on substituted acrylamide gels with those found by fitting swelling diagrams on biocompatible gels, and (iii) to discuss characteristic features of the equilibrium water uptake by biocompatible gels. The latter is of primary importance for the design of TR gels for particular biomedical applications [21,22,23].

The following scenario is widely accepted for the description of temperature-induced changes in the micro-structure of TR gels, see in [24,25] and the reviews in [26,27]. Each chain in the polymer network is presumed to consist of hydrophilic and hydrophobic segments. When temperature *T* remains below VPTT, hydrophobic segments are surrounded by cage-like structures formed by water molecules bridged by hydrogen bonds. An increase in temperature leads to the growth of intensity of thermal fluctuations that destabilize the cage-like structures. Their breakage causes “release” of hydrophobic segments for direct contact with water molecules. As a result, the overall hydrophobicity of the network (characterized by the Flory–Huggins (FH) parameter χ) increases with *T*. When molar fraction of “released” hydrophobic segments reaches its critical value (this state is characterized by the equality of the FH parameter χ to its ultimate value $\chi_{\max}$), these segments begin to aggregate into hydrophobic clusters from which water molecules are expelled. The temperature at which the aggregation process starts is identified as the volume phase transition temperature $T_{c}$. As all cage-like structures formed by water molecules around hydrophobic segments are broken above VPTT, further growth of hydrophobicity of the network does not occurs, and the FH parameter χ remains equal to its ultimate value $\chi_{\max}$.

When hydrophobic interactions between segments are strong, VPTT of a TR hydrogel coincides with the cloud point temperature of a dilute solution of polymer chains, and aggregation of hydrophobic segments into clusters occurs within a few K (an abrupt transition from the swollen state into the collapsed state of a gel). The volume phase transition temperature $T_{c}$ is entirely determined by the balance of interactions between hydrophilic and hydrophobic segments [28]. The equilibrium degree of swelling *Q* of a gel in the collapsed state is small, and it is practically independent of molar fractions of monomers and cross-linker under preparation conditions. For example, observation on PNIPAm macroscopic gels and microgels [29,30] show that

(1) $$Q\left(T_{*}\right)=\frac{1}{2}$$

at temperatures $T_{*}$ strongly exceeding VPTT.

In TR gels with intermediate strengths of hydrophobic interactions, VPTT remains close to the cloud point temperature of a dilute solution of polymer chains, but the process of aggregation of hydrophobic segments above $T_{c}$ is affected by such factors as elasticity of the polymer network, hydrogen bonds between chains, repulsion of ionized functional groups, and van der Waals forces. Due to the influence of these factors, the gels demonstrate multi-step aggregation processes [31], and their equilibrium degrees of swelling above VPTT adopt relatively high values (up to 10): the swollen state is transformed into a sponge-like state instead of the collapsed state [32].

When interactions between hydrophobic segments in a TR gel are weak, the gel exhibits its temperature sensitivity as a decay in the equilibrium degree of swelling with *T*, but, unlike dilute solutions of the corresponding polymer chains, does not reveal the volume phase transition (associated with formation of hydrophobic clusters). Two reasons for the disappearance of VPT can be mentioned: (i) elasticity of the polymer network that resists formation of hydrophobic clusters and hinders the aggregation process, and (ii) a strong decay in the equilibrium degree of swelling with temperature before the hydrophobicity of the network (characterized by the FH parameter χ) reaches its critical value $\chi_{\max}$. In the latter case, most water molecules are expelled from the gel before concentration of “released” hydrophobic segments becomes critical, and the driving force for aggregation disappears.

The aim of the present work is to show that the above three scenarios are realized in biocompatible TR gels. In particular, (i) PVCL gels with strong hydrophobic interactions exhibit equilibrium swelling diagrams similar to those of PNIPAm gels and (ii) PVME and PDMAEMA gels (with hydrophobic interactions of intermediate strengths) reveal transition from the swollen to the sponge-like state at the critical temperature $T_{c}$, whereas (iii) poly(2-oxazoline)s and poly(methoxyethoxy ethyl methacrylate)s demonstrate temperature-sensitivity without volume phase transition (aggregation of hydrophobic segments does not occur in these gels).

## 2. Model

### 2.1. Swelling on Homopolymer Gels

A thermo-responsive homopolymer gel is modeled as a two-phase medium composed of an equivalent polymer network and water molecules. The solid and fluid phases are thought of as immiscible interpenetrating continua.

The initial state of a gel coincides with that of an undeformed dry specimen at some temperature $T_{0}<T_{c}$. Transformation of the initial state into the actual state at temperature *T* is described by the deformation gradient F that obeys the molecular incompressibility condition

(2) detF=1+Q,

where det is the determinant, Q=Cv is the degree of swelling, *C* denotes concentration of water (number of molecules per unit volume in the initial state), and *v* is the characteristic volume of water molecule.

The polymer network in a TR gel involves two components. The first (covalent) sub-network is formed under cross-linking polymerization of a solution of monomers. The other network with physical bonds is formed at temperatures $T>T_{c}$ due to aggregation of hydrophobic segments of chains. To simplify the analysis, both networks are treated as permanent. Adopting the affinity hypothesis, we suppose that deformations of the sub-networks coincide with macro-deformation of the gel.

The deformation gradient for macro-deformation F is connected with the deformation gradient for elastic deformation of the *m*th (m=1,2) network $F_{e}^{\left(m\right)}$ by the multiplicative decomposition formula

(3) $$F=F_{e}^{\left(m\right)}\cdot f_{m},$$

where $f_{m}$ is the deformation gradient for transition from the initial to the reference (stress-free) state of the *m*th network, and the dot stands for inner product.

Homogeneous transformation of the covalently cross-linked network from its initial state into the reference state is determined by the deformation gradient

(4) $$f_{1}={\left(1+Q_{0}\right)}^{\frac{1}{3}}I,$$

where I is the unit tensor, $Q_{0}=C_{0}v$ is the degree of swelling, and $C_{0}$ stands for the concentration of water molecules in the reference state.

Keeping in mind that all water molecules are expelled from hydrophobic aggregates, we presume the reference state of the network with physical bonds to coincide with the initial (dry) state of the gel,

(5) $$f_{2}=I.$$

With reference to [33], the following expression is adopted for the Helmholtz free energy of the gel (per unit volume in the initial state):

(6) $$\Psi =\Psi_{1}+\Psi_{2}+\Psi_{\mathrm{int}},$$

where $\Psi_{1}$ is the specific energy of water molecules not interacting with segments of chains, $\Psi_{2}$ is the specific energy of polymer chains not interacting with water, and $\Psi_{\mathrm{int}}$ is the specific energy of mixing of water molecules with segments of chains.

The specific energy of water molecules is given by

(7) $$\Psi_{1}=\mu^{0}C,$$

where $\mu^{0}$ is the chemical potential of water molecules not interacting with the polymer network.

The specific energy of the network (consisting of two parts with chemical and physical bonds) reads

(8) $$\Psi_{2}=\sum_{m=1}^{2}W_{m}\left(I_{e1}^{\left(m\right)},I_{e2}^{\left(m\right)},I_{e3}^{\left(m\right)}\right).$$

The specific mechanical energy $W_{m}$ stored in chains of the *m*th network depends on the principal invariants $I_{e1}^{\left(m\right)},I_{e2}^{\left(m\right)},I_{e3}^{\left(m\right)}$ of the corresponding Cauchy–Green tensor for elastic deformation

(9) $$B_{e}^{\left(m\right)}=F_{e}^{\left(m\right)}\cdot F_{e}^{(m)⊤},$$

where ⊤ stands for transpose. The neo-Hookean expressions are accepted for the functions $W_{m}$,

(10) $$W_{m}=\frac{1}{2}G_{m}\left[\left(I_{e1}^{\left(m\right)}-3\right)-\ln I_{e3}^{\left(m\right)}\right],$$

where $G_{m}$ stands for the shear modulus of the *m*th network. The physical meaning of Equation (10) was discussed in [34], where this formula was re-derived within the concept of entropic elasticity. More sophisticated expressions for the functions $W_{m}$ were developed and verified by comparison with observations in [35,36].

The specific energy of mixing of water molecules with segments of chains reads [33]

(11) $$\Psi_{\mathrm{int}}=k_{B}T_{0}\left(C\ln\phi_{w}+\chi C\phi_{n}\right),$$

where $k_{B}$ is the Boltzmann constant, and

(12) $$\phi_{w}=\frac{Cv}{1+Cv},\;\phi_{n}=\frac{1}{1+Cv}$$

are volume fractions of water and polymer network in the actual state. The first term in Equation (11) characterizes the entropy and the other term describes the enthalpy of mixing of water molecules and segments of chains. In a narrow interval of temperatures near $T_{c}$, the actual temperature *T* is replaced with the initial temperature $T_{0}$ in the thermodynamic factor $k_{B}T$.

Unlike the conventional approach [37], we treat the FH parameter χ as a function of temperature only:

(13) $$\chi =\chi_{0}+\chi_{1}T\;\left(T<T_{c}\right),\;\chi =\chi_{\max}\;\left(T\geq T_{c}\right),$$

where the coefficients $\chi_{0}$, $\chi_{1}$, and $\chi_{\max}$ obey the continuity condition

(14) $$\chi_{\max}=\chi_{0}+\chi_{1}T_{c}.$$

Equation (13) means that breakage of cage-like structures formed by water molecules around hydrophobic segments leads to an increase in the effective hydrophobicity of chains (characterized by χ) at temperatures *T* below $T_{c}$. Above VPTT, the growth of χ is prohibited due to formation of aggregates of hydrophobic segments. These conditions ensure that material parameters do not accept anomalously high values mentioned in [38].

With reference to the Landau theory of phase transition, volume phase transition in a TR gel is characterized by the order parameter η:

(15) $$\eta =0\;\left(T<T_{c}\right),\;\eta =\chi -\chi_{\max}\;\left(T\geq T_{c}\right),$$

where χ is given by Equation (13). Equation (15) differs from the expression for the order parameter proposed in [39,40].

The elastic modulus of the covalently cross-linked network $G_{1}$ is presumed to be independent of temperature. The modulus $G_{2}$ of the network with physical bonds vanishes below $T_{c}$ (when the gel is in the swollen state) and grows with the order parameter above $T_{c}$ (when the gel is in the collapsed state) due to formation of hydrophobic clusters that serve as extra physical bonds between chains. The increase in $G_{2}$ is described by the differential equation

(16) $$\frac{dG_{2}}{d\eta }=\left(\beta +\beta_{1}\eta \right)\left({\bar{G}}_{2}-G_{2}\right),\;G_{2}\left(0\right)=0,$$

where ${\bar{G}}_{2}$, β, and $\beta_{1}$ are material constants. Taking into account that $G_{2}$ is proportional to concentration hydrophobic clusters that bridge polymer chains in the collapsed state, Equation (16) is treated as the governing equation for aggregation of hydrophobic segments.

Under unconstrained swelling of a TR gel at an arbitrary temperature *T*, its equilibrium degree of swelling *Q* obeys the nonlinear equation

(17) $$\ln\frac{Q}{1+Q}+\frac{1}{1+Q}+\frac{\chi }{{\left(1+Q\right)}^{2}}+\frac{g_{1}}{1+Q}\left[{\left(\frac{1+Q}{1+Q_{0}}\right)}^{\frac{2}{3}}-1\right]+\frac{g_{2}}{1+Q}\left[{\left(1+Q\right)}^{\frac{2}{3}}-1\right]=0,$$

where

(18) $$g_{m}=\frac{G_{m}v}{k_{B}T_{0}}\;\left(m=1,2\right)$$

are dimensionless elastic moduli. Derivation of Equation (17) based on the free energy imbalance inequality is given in [41].

Equation (17) together with Equation (13) for the FH parameter χ, Equation (15) for the order parameter η, and Equations (16) and (18) for the dimensionless modulus $g_{2}$ provide governing equations for the equilibrium degree of swelling *Q* of a TR gel. These relations involve eight adjustable parameters: (i) the coefficients $\chi_{0}$ and $\chi_{1}$ describe an increase in hydrophobicity of chains with temperature below VPTT; (ii) $\chi_{\max}$ characterizes their hydrophobicity above VPTT; (iii) $g_{1}$ and $Q_{0}$ stand for the dimensionless shear modulus of the covalently cross-linked network and its degree of swelling in the reference state, respectively; and (iv) the parameters ${\bar{g}}_{2}$, β, and $\beta_{1}$ describe aggregation of hydrophobic segments above VPTT. The volume phase transition temperature $T_{c}$ is determined by Equation (14). The parameter $\beta_{1}$ is introduced into the model to describe equilibrium swelling curves on PVCL gels. For the other gels under consideration, this coefficient vanishes.

### 2.2. Swelling of Copolymer Gels

#### 2.2.1. Gels with Strong Hydrophobic Interactions

We begin with the analysis of TR gels prepared by cross-linking copolymerization of thermo-responsive monomers (with hydrophobic interactions of high and intermediate strengths) and nonionic temperature-insensitive monomers. Molar fractions of temperature-insensitive and thermo-responsive monomers in the feed equal ψ and 1−ψ, respectively, where ψ is small compared with unity,

(19) ψ≪1.

The FH parameter of a copolymer gel χ is determined by the formula [42,43]

(20) $$\chi =\chi_{\mathrm{TR}}\left(1-\psi \right)+\chi_{\mathrm{TI}}\psi -\chi_{\mathrm{INT}}\psi \left(1-\psi \right),$$

where $\chi_{\mathrm{TR}}$ and $\chi_{\mathrm{TI}}$ are the FH parameters describing interactions of water molecules with thermo-responsive and temperature-insensitive monomers, respectively, and the coefficient $\chi_{\mathrm{INT}}$ characterizes binary interactions between the comonomers. Under condition (19), Equation (20) is simplified:

(21) $$\chi =\chi_{\mathrm{TR}}\left(1-\psi \right)+\tilde{\chi }\psi ,$$

where $\chi_{\mathrm{TR}}$ is given by Equation (13), and

$$\tilde{\chi }=\chi_{\mathrm{TI}}-\chi_{\mathrm{INT}}$$

stands for the modified FH parameter of comonomers (that accounts for their interactions with TR monomers and water molecules simultaneously). Bearing in mind that comonomers are temperature-insensitive, we presume $\tilde{\chi }$ to be independent of temperature as well. It follows from Equations (13) and (21) that the FH parameter of a copolymer gel reads

(22) $$\begin{array}{ccc} & & \chi =\left[\chi_{0}\left(1-\psi \right)+\tilde{\chi }\psi \right]+\chi_{1}\left(1-\psi \right)T\;\left(T<T_{c}\right),\\ & & \chi =\chi_{\max}\;\left(T\geq T_{c}\right). \end{array}$$

The coefficients $\chi_{0}$, $\chi_{1}$ in Equation (22) are treated as “universal” constants for a TR gel, which means that they are determined uniquely by the chemical structure of monomers and are independent of molar fraction of cross-linker and preparation conditions. The parameter $\chi_{\max}$ is presumed to be “semi-universal”, which implies that it may be affected by conditions of synthesis, but remains independent of chemical structure and molar fraction of comonomers. According to Equation (22), VPTT of a copolymer gel is given by

(23) $$T_{c}=\frac{\chi_{\max}-\chi_{0}}{\chi_{1}}\frac{1+a\psi }{1-\psi },\;a=\frac{\chi_{0}+\tilde{\chi }}{\chi_{\max}-\chi_{0}}.$$

We suppose that the kinetics of aggregation of hydrophobic segments above VPTT is not affected by the presence of temperature-insensitive comonomers. This means that the coefficients ${\bar{g}}_{2}={\bar{G}}_{2}v/\left(k_{B}T_{0}\right)$, β, and $\beta_{1}$ are independent of ψ.

With reference to the conventional scaling rule [44], according to which the elastic modulus of a gel is uniquely determined by concentration of covalent cross-links, we treat $g_{1}$ as a coefficient independent of ψ.

Bearing in mind that degree of swelling in the reference (stress-free) state $Q_{0}$ is influenced by the effective hydrophilicity of monomers in the feed [44], we presume $Q_{0}$ to evolve with molar fraction of comonomers ψ. Introducing volume fraction of polymer network in the reference state $\phi_{n0}$ (this quantity is determined by Equation (12) where *C* is replaced with $C_{0}$), we describe the effect of ψ on $\phi_{n0}$ by the linear equation

(24) $$\phi_{n0}=\phi_{n0}^{0}+\phi_{n0}^{1}\psi ,$$

where $\phi_{n0}^{0}$ and $\phi_{n0}^{1}$ are material parameters.

#### 2.2.2. Gels with Weak Hydrophobic Interactions

A characteristic feature of the thermo-mechanical response of gels with weak hydrophobic interactions is that their FH parameter χ grows with temperature *T* rather feebly. To reduce the number of adjustable parameters in the model and to ensure stability of the fitting algorithm, we set

(25) $$\chi_{0}=0$$

in Equation (13), which implies that

(26) $$\chi_{\mathrm{TR}}=\chi_{1}T\;\left(T<T_{0}\right)$$

for a homopolymer gel.

It follows from Equations (20) and (26) that for a copolymer gel with an arbitrary molar fraction ψ of temperature-insensitive comonomers, the FH parameter increases linearly with temperature,

(27) $$\chi =\tilde{\chi }\psi +\chi_{1}\left(1-\psi \right)T,$$

where the effective FH parameter $\tilde{\chi }$ characterizes interactions of comonomers with TR monomers and water molecules.

As it will be shown in Section 3, TR homopolymer gels with weak hydrophobic interactions do not exhibit volume phase transition, which means that condition (26) is fulfilled in the entire interval of temperatures *T* between 0 and 100 °C. However, their copolymers with hydrophobic temperature-insensitive monomers reveal VPT, and their volume phase transition temperature $T_{c}$ is determined by Equation (14). Unlike TR gels with strong hydrophobic interactions, for which $\chi_{\max}$ is independent of chemical structure and molar fraction of comonomers, for gels with weak hydrophobic interactions, this parameter is affected by composition of comonomers. Changes in $\chi_{\max}$ with ψ are described by the relation

(28) $$\chi_{\max}=\chi_{\max}^{0}+\chi_{\max}^{1}\psi ,$$

where $\chi_{\max}^{0}$ and $\chi_{\max}^{1}$ are material parameters.

Another characteristic feature of TR copolymer gels with weak hydrophobic interactions is that their elastic moduli (below and above VPTT) increase with molar fraction of hydrophobic comonomers. To explain the growth of $g_{1}$ with ψ, we suppose that some hydrophobic clusters are developed under preparation conditions (due to the presence of hydrophobic monomers), and they serve as extra physical cross-links between chains even when copolymer gels remains in the swollen state. The increase in ${\bar{g}}_{2}$ is explained by the fact that these clusters serve as seeds in the aggregation process above VPTT. Changes in $g_{1}$ and ${\bar{g}}_{2}$ with ψ are described by the relations (the logarithmic rule of mixture)

(29) $$\log g_{1}=g_{1}^{0}+g_{1}^{1}\psi ,\;\log{\bar{g}}_{2}={\bar{g}}_{2}^{0}+{\bar{g}}_{2}^{1}\psi ,$$

where $\log=\log_{10}$, and $g_{1}^{0}$, $g_{1}^{1}$, ${\bar{g}}_{2}^{0}$, ${\bar{g}}_{2}^{1}$ are material parameters.

We now consider equilibrium swelling of gels prepared by copolymerization of TR monomers with weak hydrophobic interactions and TR comonomers with weak or intermediate hydrophobic interactions. The FH parameters of the comonomers are denoted as $\chi^{\left(1\right)}$ and $\chi^{\left(2\right)}$. Bearing in mind that the use of Equation (20) is worthless in this case (as the coefficient $\chi_{\mathrm{INT}}$ accounting for mutual interactions between monomers depends on two variables: *T* and ψ), the effect of molar fraction of comonomers ψ on the FH parameter of a copolymer gel is predicted by means of the Maxwell–Garnett mixing rule. With reference to this relation (which is conventionally employed in the micro-mechanical analysis of physical properties of composites [45]), we assume that

(30) $$\chi =\frac{\chi^{\left(1\right)}\left(1-\psi \right)+A\chi^{\left(2\right)}\psi }{(1-\psi )+A\psi },$$

where *A* is a temperature-independent adjustable parameter. Equation (30) ensures that $\chi =\chi^{\left(1\right)}$ and $\chi =\chi^{\left(2\right)}$ in the limiting cases ψ=0 and ψ=1, respectively, and it is transformed into Equation (21) when A=1.

It will be shown in Section 3 that these TR copolymer gels do not reveal the volume phase transition (which means that χ increases linearly with temperature *T* in the entire interval of temperatures between 0 and 100 °C, and aggregation of hydrophobic segments is not observed). The equilibrium swelling curve on a copolymer gel with an arbitrary ψ is characterized by three parameters: the coefficient $\chi_{1}$ in Equation (26), the elastic modulus $g_{1}$, and the volume fraction of polymer network in the reference state $\phi_{n0}$. The elastic modulus $g_{1}$ is independent of ψ, the parameter $\chi_{1}$ is determined by Equation (30), and evolution of $\phi_{n0}$ with ψ is governed by the relation (an analog of Equation (30))

(31) $$\phi_{n0}=\frac{\phi_{n0}^{\left(1\right)}\left(1-\psi \right)+B\phi_{n0}^{\left(2\right)}\psi }{(1-\psi )+B\psi },$$

where *B* is an adjustable parameter.

## 3. Fitting of Experimental Data

To evaluate the ability of the model to describe observations, we approximate experimental swelling diagrams on homo- and copolymer gels with strong, intermediate, and weak hydrophobic interactions.

### 3.1. *N*-Substituted Polyacrylamide Homo- and Copolymer Gels

**Figure 1:**
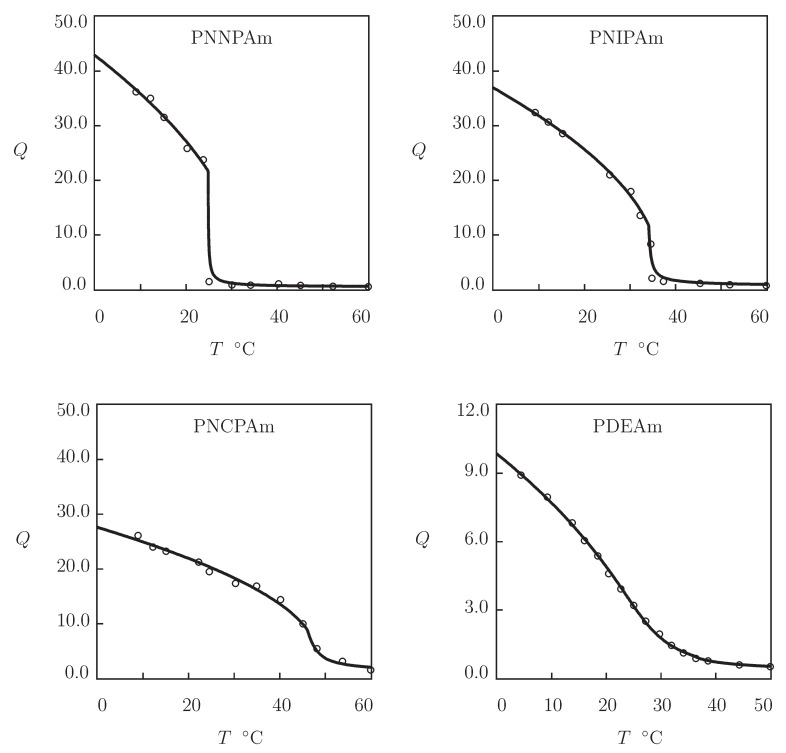
Degree of swelling *Q* versus temperature *T*. Circles: experimental data on poly(N,n-propylacrylamide) (PNNPAm), poly(*N*-isopropylacrylamide) (PNIPAm), poly(*N*-cyclopropylacrylamide) (PNCPAm) [12], and poly(N,N-diethylacrylamide) (PDEAm) [46] gels. Solid lines: results of simulation.

We begin with the analysis of observations in equilibrium water uptake tests on four *N*-substituted polyacrylamide gels (Figure 1). These data are used as a benchmark for the assessment of swelling properties of biocompatible TR gels.

Equilibrium swelling diagrams on poly(N,n-propylacrylamide) (PNNPAm), poly(*N*-isopropylacrylamide) (PNIPAm), and poly(*N*-cyclopropylacrylamide) (PNCPAm) gels are reported in Figure 1A–C, respectively. The gels were prepared by free radical polymerization (24 h at room temperature) of aqueous solutions of monomers (molar fraction of monomers 0.7 M) by using $N,N^{\prime }$-methylenebisacrylamide (BIS, molar fraction 25 mM) as a cross-linker, potassium persulfate (KPS) as an initiator, and $N,N,N^{\prime },N^{\prime }$-tetramethylethylenediamine (TEMED) as an accelerator (Inomata et al. [12]). Observations on poly(N,N-diethylacrylamide) (PDEAm) gel are presented in Figure 1D. The gel was synthesized by γ-irradiation (irradiation dose 84 kGy at room temperature) of an aqueous solution of PDEAm chains (number-average molecular weight 8.2 kg/mol, polydispersity index 1.6) (Kishi et al. [46]).

Each set of data in Figure 1 is fitted separately by means of the following procedure. At the first step, observations are approximated below VPTT (where $g_{2}=0$). The coefficient $Q_{0}$ is found from the condition that the reference state of a gel coincides with its as-prepared state [44]. The experimental dependence χ(T) is determined from Equation (17). The data are matched by Equation (13), where $\chi_{0}$ and $\chi_{1}$ are calculated by the least-squares technique. The modulus $g_{1}$ is found from the best-fit condition. At the other step, observations above VPTT are matched by Equation (16) with $\beta_{1}=0$. The parameters β and ${\bar{g}}_{2}$ are determined by the nonlinear regression method to minimize the expression $\sum {\left(Q_{\exp}-Q_{\mathrm{sim}}\right)}^{2}$, where summation is performed over all temperatures *T* under consideration, $Q_{\exp}$ stands for the degree of swelling measured in a test, and $Q_{\mathrm{sim}}$ is determined from Equation (17) that is solved by the Newton–Raphson algorithm.

Figure 1 demonstrates good agreement between the experimental data and results of simulation with the material parameters collected in Table S1. This table shows that the higher the hydrophilicity of the monomers, the larger the coefficient $\chi_{1}$ and the smaller the values of parameters $\chi_{0}$ and $\chi_{\max}$. The coefficients ${\bar{g}}_{2}$ and β (that describe the kinetics of aggregation of hydrophobic segments above VPTT) adopt similar values for all gels under consideration.

**Figure 2:**
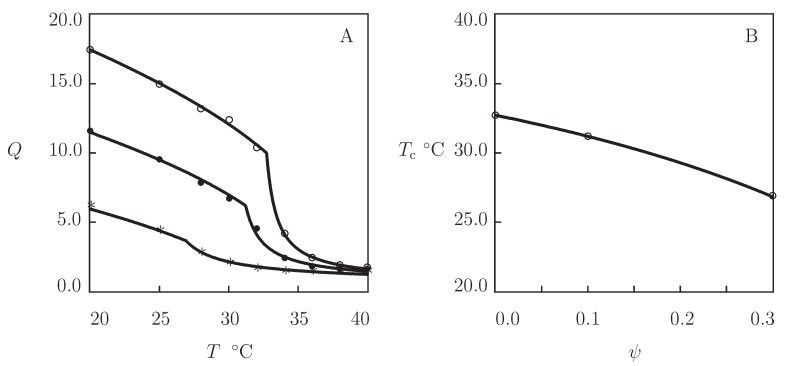
(**A**) Degree of swelling *Q* versus temperature *T*. Symbols: experimental data [47] on P(NIPAm-HEMA) gels with various molar fractions of HEMA monomers ψ=0 (∘), ψ=0.1 (•), and ψ=0.3 (*). Solid lines: results of simulation. (**B**) Volume phase transition temperature $T_{c}$ versus molar fraction of HEMA monomers ψ. Circles: experimental data. Solid line: results of simulation.

As an example of observations on copolymer gels, experimental data in equilibrium swelling tests on poly(*N*-isopropylacrylamide-co-2-hydroxyethyl methacrylate) (P(NIPAm-HEMA)) gels are presented in Figure 2. The gels were prepared by cross-linking polymerization (24 h at room temperature) of a solution of monomers (total volume fraction of monomers in solution 0.5, molar fraction of HEMA monomers ranged from 0 to 0.3) in 1:1 (**v/v**) mixture of water and acetone by using BIS (molar fraction with respect to monomers 0.03) as a cross-linker, APS as an initiator, and TEMED as an accelerator (Lee and Huang [47]). Observations on the copolymer gels with ψ=0, 0.1, and 0.3 are depicted in Figure 2A together with results of simulation with the material parameters collected in Table S2. This table shows that the quantities $\chi_{\max}$, $g_{1}$, ${\bar{g}}_{2}$ and β are independent of molar fraction of comonomers, the coefficients $\chi_{0}$ and $\chi_{1}$ evolve with ψ following Equation (21) with $\tilde{\chi }=1.564$, and $\phi_{n0}$ increases strongly with ψ (as HEMA is more hydrophobic than NIPAm). The influence of ψ on volume fraction of polymer network in the reference state $\phi_{n0}$ is illustrated in Figure S1, where the data and their approximation by Equation (24) are reported. The effect of molar fraction of comonomers on VPTT of P(NIPAm-HEMA) gel is shown in Figure 2B, where observations are plotted together with predictions of Equation (23).

### 3.2. Poly(*N*-Vinyl Caprolactam) Homo- and Copolymer Gels

Poly(*N*-vinyl caprolactam) (PVCL) gels are considered as a valuable alternative to PNIPAm gels in biomedical applications [48,49]. Their volume phase transition temperature is close to that for PNIPAm gels. Unlike PNIPAm, PVCL is bioinert (stable against hydrolysis), biocompatible [14], and it forms complexes with proteins [50]. Two characteristic features of poly(*N*-vinyl caprolactam) are worth mentioning: (i) its low critical solution temperature is strongly affected by molecular weight $M_{w}$ of polymer chains (the critical temperature $T_{c}$ is reduced by 12 K when $M_{w}$ grows from 9 to 275 kg/mol) [51], and (ii) formation of hydrophobic clusters in PVCL gels demonstrates two-stage kinetics with hydrogen bonding between segments predominating at the initial stage and hydrophobic interaction playing the key role at the final stage [31].

**Figure 3:**
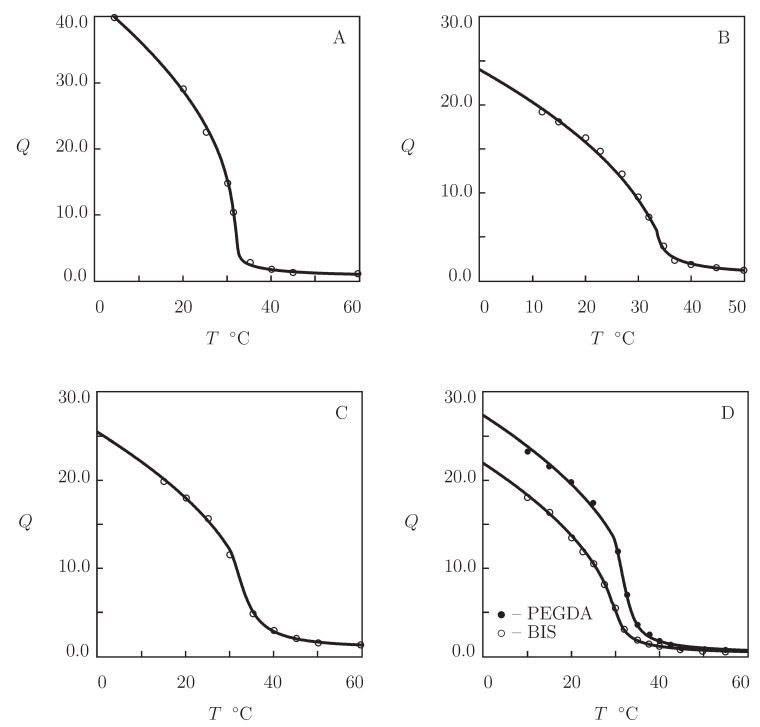
Degree of swelling *Q* versus temperature *T*. Symbols: experimental data on PVCL macro- and microgels ((**A**) [52], (**B**) [53], (**C**) [54], and (**D**) [55]). Solid lines: results of simulation.

Four sets of experimental data on PVCL homopolymer gels are matched (Figure 3). Figure 3A presents observations on PVCL gel prepared by free radical cross-linking polymerization (at 60 °C) of a solution of VCL monomers (volume fraction 0.5) in a mixture of ethanol and water by using BIS (mass fraction 0.0002) as a cross-linker and 2,2${}^{\prime }$-azobis(2-methylpropionitrile) (AIBN) as an initiator (Makhaeva et al. [52]). The experimental swelling diagram in Figure 3B is obtained on PVCL microgel synthesized by emulsion polymerization (14 h at 70 °C) of an aqueous solution of VCL monomers (mass fraction 0.43) by using BIS (mass fraction with respect to monomers 0.03) as a cross-linker, sodium dodecyl sulfate (SDS; mass fraction 0.05) as a surfactant, and KPS as an initiator (Liu et al. [53]). Figure 3C reports experimental data on PVCL microgels prepared by emulsion polymerization (20 h at 75 °C) of an aqueous solution of VCL monomers (mass fraction 0.0067) by using BIS (mass fraction with respect to monomers 0.02) as a cross-linker, poly(ethylene oxide) macromonomer (PEO-R-MA, mass fraction with respect to monomers 0.44) as a nonionic surfactant, and 2,2${}^{\prime }$-azobis[2-methyl-*N*-(2-hydroxyethyl)propionamide] (VA-086) as an initiator (Laukkanen et al. [54]). In Figure 3D, observations are presented on two PVCL microgels synthesized by emulsion polymerization (5 h at 70 °C) of an aqueous solution of VCL monomers (mass fraction 0.01) by using BIS and poly(ethylene glycol) diacrylate (PEGMA-200) as cross-linkers (molar fraction with respect to monomers 0.036), SDS as a surfactant (mass fraction 0.01), and KPS as an initiator (Imaz and Forcada [55]).

The equilibrium degree of swelling of a microgel Q(T) is calculated from the equation

(32) $$\frac{1+Q(T)}{1+Q(T_{*})}={\left(\frac{r_{h}\left(T\right)}{r_{h}\left(T_{*}\right)}\right)}^{3},$$

where $r_{h}\left(T\right)$ stands for the hydrodynamic radius measured at temperature *T*, $T_{*}$ is the maximum temperature at which observations are provided, and $Q(T_{*})$ is given by Equation (1).

**Figure 4:**
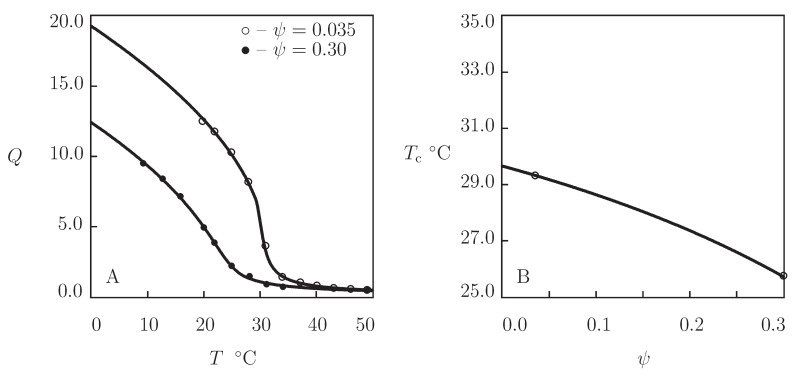
(**A**) Degree of swelling *Q* versus temperature *T*. Symbols: experimental data [56] on P(VCL-MEA) microgels with various molar fractions ψ of MEA monomers. Solid lines: results of simulation. (**B**) Volume phase transition temperature $T_{c}$ versus molar fraction of MEA monomers ψ. Circles: treatment of experimental data. Solid line: results of simulation.

Figure 3 shows an acceptable good agreement between the experimental swelling diagrams and results of simulation with the material parameters listed in Table S3. This table reveals that (i) the data are fitted correctly by the model with “universal” coefficients $\chi_{0}$ and $\chi_{1}$; (ii) the parameter $\chi_{\max}$ is practically independent of preparation conditions and molar fractions of monomers and cross-linker; (iii) the coefficients $g_{1}$ and $Q_{0}$ adopt similar values for all gels under consideration; (iv) the modulus ${\bar{g}}_{2}$ of microgels exceeds strongly that of the macroscopic gel; and (v) the coefficient β is small (compared with PNIPAm gels), whereas $\beta_{1}$ adopts relatively large values (which implies that hydrophobic interactions between segments play the key role in the aggregation process). Numerical analysis evidences that VPTTs of microgels cross-linked with BIS and PEGMA (Figure 4) coincide practically, which means that visual determination of $T_{c}$ for hydrogels with continuous volume phase transitions may lead to relatively large discrepancies.

To examine the ability of the model to describe equilibrium swelling of PVCL copolymer gels, we focus on observations on poly(*N*-vinylcaprolactam-co-2-methoxyethyl acrylate) P(VCL-MEA) microgels (Figure 4).

Microgel particles were prepared by free radical precipitation polymerization (7 h at 70 °C) of aqueous solutions on monomers (mass fraction of monomers 0.013, molar fraction of MEA monomers ψ=0.035 and 0.3) by using BIS (molar fraction with respect to monomers 0.026) as a cross-linker and 2,2${}^{\prime }$-azobis(2-methylpropyonamidine)dihydrochloride (AMPA) as an initiator (Melle et al. [56]). Figure 4A shows good agreement between the experimental swelling diagrams and results of simulation with the material parameters reported in Table S4. Bearing in mind that MEA is hydrophobic at all temperatures T≥0 °C, the coefficients $\chi_{0}$ and $\chi_{1}$ are determined from Equation (21) with $\tilde{\chi }=1.0705$. Evolution of the volume phase transition temperature $T_{c}$ with molar fraction ψ of MEA monomers is illustrated in Figure 4B, where results of numerical analysis are compared with predictions of Equation (23).

### 3.3. Poly(Methyl Vinyl Ether) Homopolymer Gels

Poly(vinyl methyl ether) (PVME) is a thermo-responsive polymer that undergoes volume phase transition close to the physiological temperature. Hydrogels prepared by cross-linking of commercially available poly(methyl vinyl ether-co-maleic anhydride) chains (Gantrez AN-139) with poly(ethylene glycol) demonstrate good biocompatibility [57]. Among biomedical applications of PVME gels, it is worth mentioning (i) micro-needle arrays for transdermal drug delivery [57], (ii) sustained delivery of hydrophobic drugs [58], and (iii) 3D scaffolds for cell growth [59].

**Figure 5:**
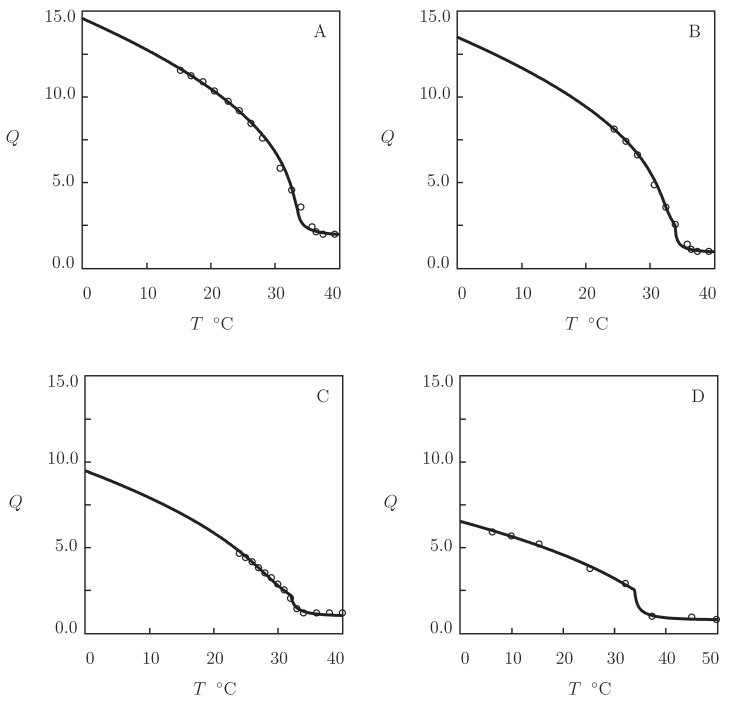
Degree of swelling *Q* versus temperature *T*. Circles: experimental data on PMVE gels ((**A**) [32], (**B**) [60], (**C**) [61], and (**D**) [62]). Solid lines: results of simulation.

Equilibrium swelling diagrams on PVME gels are reported in Figure 5 together with results of numerical simulation with the material parameters collected in Table S5. Experimental data in Figure 5A are obtained on PVME gel prepared by irradiation of an aqueous solution of PMVE (mass-average molecular weight 46 kg/mol, concentration 4 g/L) by an electron beam with the dose 80 kGy at temperature 60 °C (Arndt et al. [32]). Figure 5B presents observations on PVME gel prepared by irradiation of an aqueous solution of PVME (mass-average molecular weight 13.9 kg/mol, polydispersity index 2.4, and concentration 200 g/L) by an electron beam with the dose 80 kGy at room temperature (Richter [60]). In Figure 5C, an experimental swelling diagram is reported on PVME gel film prepared by spin-coating of a solution of PVME (mass-average molecular weight 57 kg/mol, concentration 0.03) in a mixture of water and ethanol onto a silicon substrate subjected to irradiation by an electron beam with the dose 250 kGy (Hegewald et al. [61]). Figure 5D presents observations on PVME composite gel (reinforced with ferric oxide powder) prepared by γ-irradiation of an aqueous solution of PVME (mass-average molecular weight 90 kG/mol, concentration 0.3) and Fe${}_{3}$O${}_{4}$ particles (concentration 0.15) with the dose 110 kGy at room temperature (Kabra et al. [62]).

Figure 5 confirms the ability of the model with “universal” coefficients $\chi_{0}$ and $\chi_{1}$ to describe equilibrium swelling of PVME gels. The coefficients $\chi_{\max}$ coincide practically for all gels under consideration. The parameters $g_{1}$, $Q_{0}$, ${\bar{g}}_{2}$, and β adopt similar values for neat PVME gels (Figure 5A–C), while the moduli $g_{1}$ and ${\bar{g}}_{2}$ are slightly higher for the composite gel (Figure 5D). These results evidence that equilibrium swelling of PVME gels is weakly affected by molecular weight of polymer chains, their concentration in aqueous solutions, type of irradiation (electron beam versus γ-irradiation), and irradiation dose.

PVME gels are characterized by rather large (exceeding 2) values of the equilibrium degree of swelling above VPTT: unlike PNIPAm gels that expel water molecules in the collapsed state (Equation (1)), PVME gels form sponge-like structures at $T>T_{c}$ [32]. This is reflected in the model by low values of the elastic modulus ${\bar{g}}_{2}$ (this parameter in Table S1 exceeds ${\bar{g}}_{2}$ in Table S5 by an order of magnitude).

### 3.4. Poly(N,N-Dimethylaminoethyl Methacrylate) Homo- and Copolymer Gels

Poly(N,N-dimethylaminoethyl methacrylate) (PDMAEMA) gels are cationic hydrogels (due to the presence of tertiary amines) with the dissociation constant ${\mathrm{pK}}_{a}$ close to 8.5 [63]. Due to the ionic nature of these materials, they are sensitive to temperature, pH, and molar fraction of salts in aqueous solutions [64]. A particular interest to PDMAEMA-based gels is driven by their antibacterial activity [65] and applications as non-viral gene delivery systems [66,67]. Although PDMAEMA is a polymer with relatively high cytotoxicity [68,69], toxicity of PDMAEMA gels is strongly reduced by copolymerization of DMAEMA monomers with biocompatible monomers [70,71,72,73].

As this study focuses on modeling the response of nonionic TR gels, we confine ourselves to the analysis of observations on PDMAEMA gels obtained under similar experimental conditions.

**Figure 6:**
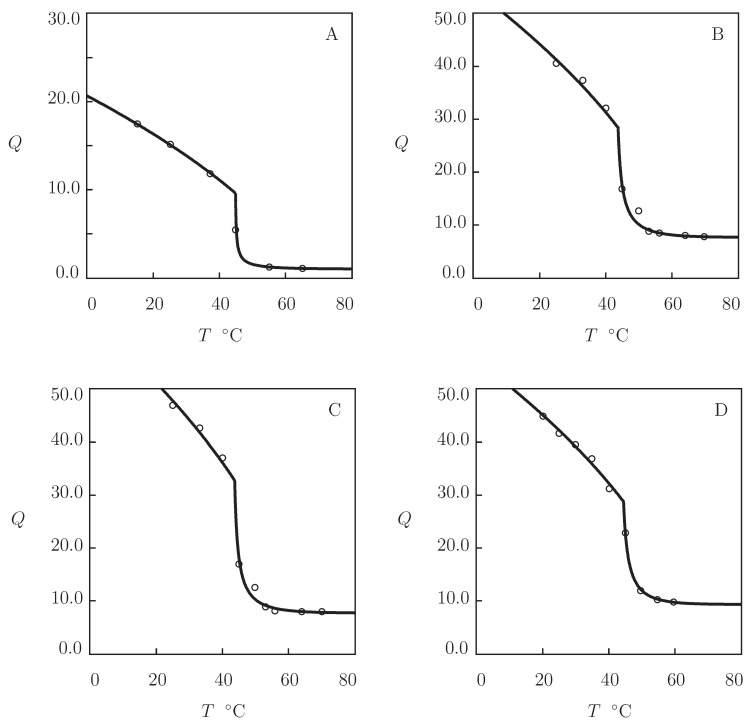
Degree of swelling *Q* versus temperature *T*. Circles: experimental data on PDMAEMA gels ((**A**) [74], (**B**) [75], (**C**) [76], and (**D**) [77]). Solid lines: results of simulation.

Equilibrium swelling diagrams on four PDMAEMA homopolymer gels are presented in Figure 6. Data in Figure 6A were obtained in equilibrium swelling tests on PDMAEMA gel prepared by free-radical cross-linking polymerization (24 h at 60 °C) of a solution of DMAEMA monomers (mass fraction 0.2 g/L) in a 1:9 (*v/v*) mixture of water and isobutanol by using ethylene glycol dimethacrylate (EGDMA; molar fraction with respect to monomers 0.005) as a cross-linker and $N,N^{\prime }$-azobis(isobutyronitrile) (AIBN) as an initiator. Tests were performed in a phosphate buffer with pH=8 and ionic strength 0.1 M (Emileh et al. [74]).

Figure 6B reports observations in swelling tests on PDMAEMA gel synthesized by γ-irradiation at room temperature (the total irradiation dose was not provided) of an aqueous solution of monomers (molar fraction 1 M) and poly(ethylene glycol dimethacrylate) (PEGDMA, molecular weight $M_{w}=875$) as a cross-linker. Equilibrium water uptake tests were conducted in deionized water (Li et al. [75]).

Figure 6C presents experimental data on PDMAEMA gel prepared and tested by means of a similar procedure (Li et al. [76]). The only difference between observations in Figure 6B,C consists in the molar fraction of cross-linker with respect to monomers: it equals $1.2\times 10^{-4}$ for the data in Figure 6B and $3.5\times 10^{-3}$ for those in Figure 6C.

Figure 6D reports observations in equilibrium swelling tests in deionized water on PDMAEMA gel prepared by free radical cross-linking polymerization (15 h at 75 °C) of a solution of monomers (molar fraction 0.496 M) in a 1:1 (*v/v*) mixture of water and ethanol by using BIS (molar fraction with respect to monomers $1.8\times 10^{-3}$) as a cross-linker and AIBN as an initiator (Cho et al. [77]).

Figure 6 demonstrates an acceptable agreement between the experimental data and results of simulation with the material parameters listed in Table S6. This table shows that the coefficients $\chi_{0}$ and $\chi_{1}$ coincide for all gels, and $\chi_{\max}$, $g_{1}$, and β adopt similar values. The parameters ${\bar{g}}_{2}$ and $Q_{0}$ accept similar values for all materials except for the gel whose swelling diagram is reported in Figure 6A. The latter is not surprising as experiments on this gel were performed in an aqueous solution with pH close to ${\mathrm{pK}}_{a}$ and high ionic strength (where most cationic functional groups were not ionized).

Two characteristic features of PDMAEMA gels: (i) very low values of their elastic moduli in the swollen state $g_{1}$ (these values are smaller than the corresponding moduli of PNIPAm, PVCL, and PVME gels by an order of magnitude) and (ii) high (close to 10) values of *Q* in the collapsed state (caused by the hindrance of aggregation of hydrophobic segments due to the presence of ionized functional groups).

**Figure 7:**
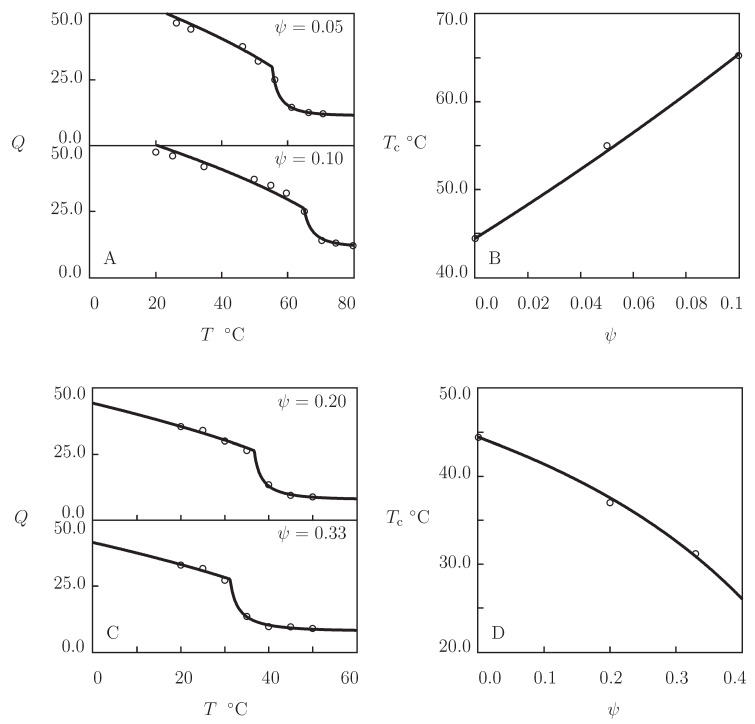
(**A**,**C**) Degree of swelling *Q* versus temperature *T*. Circles: experimental data [77] on P(DMAEMA-EAAm) (**A**) and P(DMAEMA-AAm) (**C**) gels with various molar fractions ψ of comonomers. Solid lines: results of simulation. (**B**,**D**) Volume phase transition temperature $T_{c}$ versus molar fraction ψ of comonomers. Circles: treatment of experimental data on P(DMAEMA-EAAm) (B) and P(DMAEMA-AAm) (D) gels. Solid lines: results of simulation.

Observations in equilibrium swelling tests on PDMAEMA copolymer gels are reported in Figure 7 together with results of simulation with the material parameters reported in Table S7. Experimental data on poly(N,N-dimethylaminoethyl methacrylate-co-ethyl acrylamide) P(DMAEMA-EAAm) gel are presented in Figure 7A, and those on poly(N,N-dimethylaminoethyl methacrylate-co-acrylamide) P(DMAEMA-AAm) are depicted in Figure 7C. Both gels were synthesized by free radical cross-linking polymerization (15 h at 75 °C) of solutions of comonomers (the total molar fraction of monomers 0.496 M, molar ratio ψ of EAAm monomers ranged from 0 to 0.1, and that of AAm monomers varied in the interval between 0 and 0.33) in a 1:1 (*v/v*) mixture of water and ethanol by using BIS (molar fraction with respect to monomers $1.8\times 10^{-3}$) as a cross-linker and AIBN as an initiator (Cho et al. [77]). As AAm and EAAm are temperature-insensitive, Equation (21) is used to calculate the FH parameter of copolymer gels. Changes in VPTT with molar fraction of comonomers ψ are described by Equation (23) with $\tilde{\chi }=0.68$ for hydrophobic AAm monomers and $\tilde{\chi }=-2.26$ for hydrophilic EAAm monomers. Figure 7B,D confirm that Equation (23) predicts adequately the effect of ψ on VPTT of P(DMAEMA-EAAm) and P(DMAEMA-AAm) copolymer gels.

### 3.5. Poly(2-Oxazoline) Homo- and Copolymer Gels

Poly(2-oxazoline)s (POx) form a large family of thermo-responsive polymers that have attracted substantial attention in the past decade [78,79,80,81,82]. These materials are considered as a potential alternative for PNIPAm hydrogels in biomedical applications [83] as they are highly biocompatible, non-cytotoxic [84], and non-immunogenic [85]. Although the first POx hydrogels were synthesized about 30 years ago [86], the preparation, design, and analysis of the mechanical properties of POx gels have recently become a hot topic [87].

**Figure 8:**
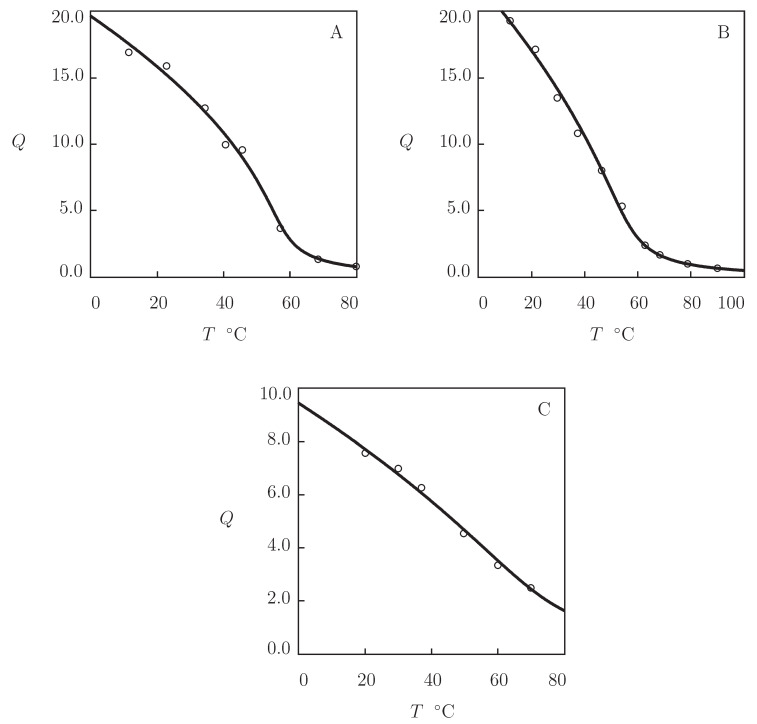
Degree of swelling *Q* versus temperature *T*. Circles: experimental data on POx gels ((**A**) PEtOx [88], (**B**) PEtOx [89], and (**C**) PIPOx [90]). Solid lines: results of simulation.

A characteristic feature of POx gels is that these materials are temperature-sensitive, but do not reveal the volume phase transition (the growth of temperature induces an increase in their overall hydrophobicity, but do not lead to formation of hydrophobic aggregates). To examine this property, three sets of experimental data on homopolymer networks are analyzed (Figure 8).

Figure 8A presents observations in equilibrium water uptake tests on poly(2-ethyl-2-oxazoline) (PEtOx) network prepared by polymerization (24 h at 120 °C) of a solution of monomers in initiator 1,4-dibromo-2-butene (DBB; monomer-to-initiator ratios: 20–200). Number-average molar mass and polydispersity index of polymer chains were estimated as $M_{n}=11.2$ kg/mol and 1.3, respectively (Christova et al. [88]).

In Figure 8B, the experimental swelling diagram is reported on PEtOx network prepared by mixture of PEtOx chains (mass-average molar mass $M_{w}=122$ kg/mol, polydispersity index 2.9) with dicumyl peroxide (DCP) as a free radical initiator and triallyl isocyanurate (TAIC) as a multi-functional cross-linker (total mass ratio of DCP and TAIC with respect to monomers 0.01, DCP to TAIC ratio 1:8) in acetone, evaporation of acetone (24 h at 80 °C), and cure of the solid mixture (30 min at 160 °C) under compression (Segiet et al. [89]).

Experimental data on poly(2-isopropenyl-2-oxazoline) (PIPOx) gel are depicted in Figure 8C. The network was prepared by mixture of PIPOx chains (number-average molar mass 10.6 kg/mol, polydispersity index 1.17) with dodecanedioic acid (mass fraction with respect to polymer 0.005) as a cross-linker in $N,N^{\prime }$-dimethylacetamide (mass fraction of polymer 0.2), followed by chemical reaction (4 h at at 140 °C) (Jerca et al. [90]).

Figure 8 shows that the experimental data are adequately described by the model with the material constants collected in Table S8. To confirm that the gels do not reveal VPT (aggregation of hydrophobic segments does not occur within the entire interval of temperatures under consideration), the experimental dependencies of the FH parameter χ on temperature *T* are reported in Figure S2 together with their approximations by Equation (26). This conclusion is also supported by observations reported in [91] (they show that the elastic modulus of PEtOx gel decreases smoothly with temperature) and in [92] (they demonstrate that the high shear rate viscosity of a similar gel increases insignificantly with temperature).

Although POx homopolymer gels do not exhibit volume phase transition, copolymerization of POx with hydrophobic monomers results in formation of networks where aggregation of segments takes place. In traditional TR gels copolymerized with hydrophobic monomers, VPTT decreases monotonically with molar fraction of comonomers ψ, and the rate of decay in $T_{c}\left(\psi \right)$ is proportional to their hydrophobicity [93]. Unlike these gels, VPTT of POx copolymer gels is practically unaffected by molar fraction and hydrophobicity of comonomers (when ψ remains sufficiently high).

**Figure 9:**
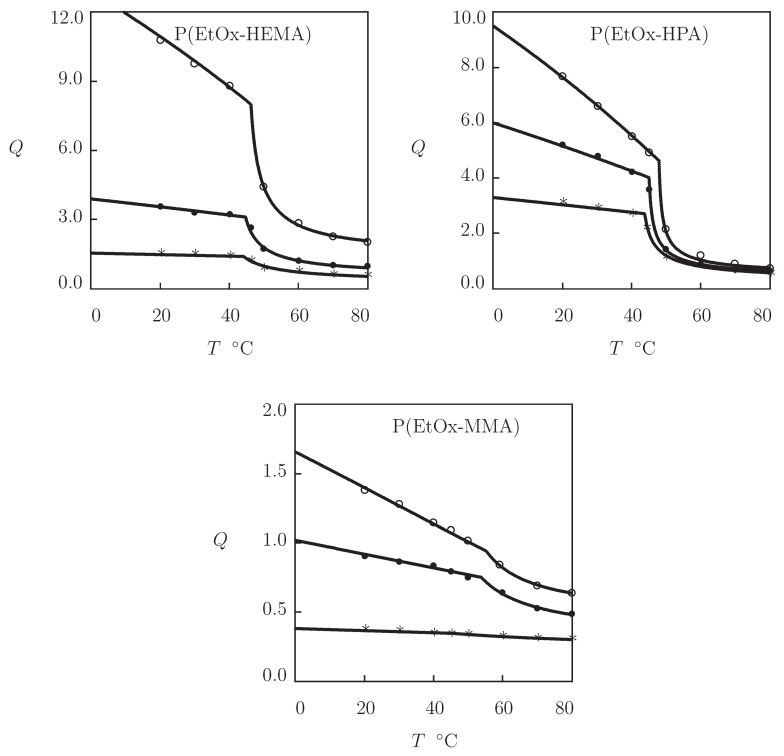
Degree of swelling *Q* versus temperature *T*. Symbols: experimental data [88] on P(EOx-HEMA), P(EOx-HPA), and P(EOx-MMA) copolymer gels with various molar fractions ψ on comonomers (∘–ψ=0.3, •–ψ=0.5, *–ψ=0.7). Solid lines: results of simulation.

To confirm this assertion, three sets of experimental data are approximated on poly(2-ethyl-2-oxazoline-co-2-hydroxyethyl methacrylate) (PEtOx-HEMA), poly(2-ethyl-2-oxazo line-co-2-hydroxypropyl acrylate) (PEtOx-HPA), and poly(2-ethyl-2-oxazoline-co-methyl meth acrylate) (PEtOx-MMA) gels (Figure 9). Copolymer networks were prepared by dissolution of EtOx-bis-macromonomers and initiator 1-methanesulfonic ester of methylbenzoine (MSMB; molar fraction with respect to comonomers 0.005) in comonomers (molar fractions ψ=0.3, 0.5 and 0.7), followed by UV irradiation (30 min with intensity 10 mW/cm${}^{2}$) and curing (24 h at 70 °C (Christova et al. [88]).

Figure 9 shows that experimental swelling curves are correctly described by the model with the material parameters collected in Table S9. The FH parameter of copolymer gels is calculated by Equation (27) with $\tilde{\chi }=-0.30$ for HEMA, $\tilde{\chi }=0.19$ for HPA, and $\tilde{\chi }=0.45$ for MMA comonomers. The volume phase transition temperature $T_{c}$ is weakly affected by ψ and the chemical structure of comonomers (it remains close to 45 °C for all gels under consideration). However, the equilibrium degree of swelling *Q* is strongly influenced by hydrophobicity of comonomers and their molar fraction both below and above the VPTT point. The effect of comonomers is accounted for by three parameters: the ultimate value of the FH parameter $\chi_{\max}$ at which aggregation of hydrophobic segments starts, and the elastic moduli $g_{1}$ and ${\bar{g}}_{2}$ characterizing concentrations of covalent and physical bonds in the swollen and collapsed states. Changes in these parameters with ψ are illustrated in Figure S3, where the data are reported together with their fits by Equations (28) and (29).

### 3.6. Poly(2-(2-Methoxyethoxy) Ethyl Methacrylate) and Poly(Oligo(Ethylene Glycol) Methyl Ether Methacrylate) Copolymer Gels

Poly(2-(2-methoxyethoxy) ethyl methacrylate) (PMEO${}_{2}$MA) and poly(oligo(ethylene glycol) methyl ether methacrylate) (POEGMA) belong to a large family of thermo-responsive polymers [94] whose biocompatibility has been confirmed in several studies [95,96]. After introduction of these materials as an alternative for PNIPAm gels [97], development of novel strategies for their synthesis and investigation of their swelling and drug release properties have become a research hotspot [98,99,100].

We begin with fitting experimental data in equilibrium water uptake tests on PMEO${}_{2}$MA and POEGMA homopolymer gels. Our aim is to show the difference between PMEO${}_{2}$MA gels (that do not reveal the volume phase transition in the interval of temperatures between 0 and 100 °C) and POEGMA gels (that demonstrate transition from the swollen to collapsed state at elevated temperatures).

**Figure 10:**
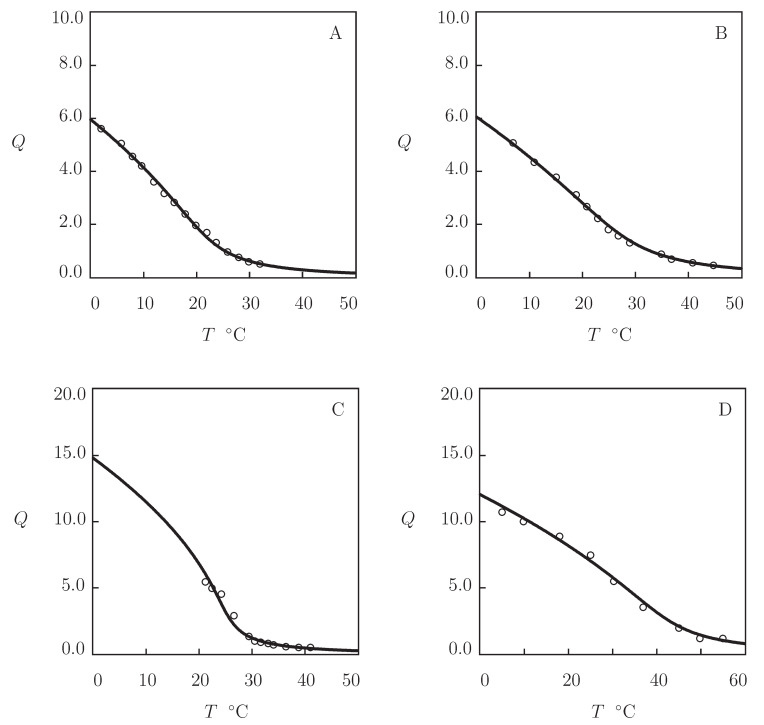
Degree of swelling *Q* versus temperature *T*. Circles: experimental data on PMEO${}_{2}$MA gels ((**A**) macroscopic gel [101], (**B**) core–shell microgel with Au core and gel shell [102], (**C**) microgel particles [103], and (**D**) nanocomposite gel [104]). Solid lines: results of simulation.

Four experimental swelling curves on PMEO${}_{2}$MA macroscopic gels and microgels are depicted in Figure 10 together with their approximations by the model with the material constants collected in Table S10.

Figure 10A presents observations on PMEO${}_{2}$MA gel prepared by cross-linking polymerization (12 h at 50 °C) of a solution of MEO${}_{2}$MA monomers (mass fraction 0.25) in 4:1 (*v/v*) mixture of water and ethanol by using EGDMA (molar fraction with respect to monomers 0.02) as a cross-linker, and KPS as an initiator (Iizawa et al. [101]).

In Figure 10B, experimental data are reported on core-shell microgels with Au core (radius $r_{c}=40$ nm) and PMEO${}_{2}$MA shell. The microgels were prepared by precipitation polymerization (2 h at 70 °C) of MEO${}_{2}$MA monomers in a colloidal dispersion of Au particles by using tri(ethylene glycol)dimethacrylate (TEGDMA, molar fraction with respect to monomers 0.05) as a cross-linker (Lapresta-Fernandez et al. [102]). Given a hydrodynamic radius $r_{h}$ of a core–shell gel, its degree of swelling *Q* is calculated from the equation

(33) $$\frac{1+Q(T)}{1+Q(T_{*})}={\left(\frac{r_{h}\left(T\right)-r_{c}}{r_{h}\left(T_{*}\right)-r_{c}}\right)}^{3}.$$

Figure 10C presents observations on PMEO${}_{2}$MA microgels synthesized by emulsion polymerization (6 h at 70 °C) of an aqueous solution of monomers by using EGDMA (molar fraction with respect to monomers 0.028) as a cross-linker, KPS as an initiator, and sodium dodecyl sulfate (SDS) as a surfactant (Cai et al. [103]).

In Figure 10D, experimental data are reported on physically cross-linked PMEO${}_{2}$MA gel prepared by free radical polymerization (24 h at room temperature) of an aqueous solution of MEO${}_{2}$MA monomers (mass fraction 0.1) and Laponite XLS nanoclay (mass fraction 0.05) by using KPS as an initiator and TEMED as an accelerator (Xia et al. [104]).

Figure 10 shows that equilibrium swelling diagrams on all PMEO${}_{2}$MA gels are adequately described by the model that presumes no volume phase transition in the interval of temperatures *T* under consideration. To confirm this conclusion, the experimental dependencies χ(T) are presented in Figure S4 together with their approximations by Equation (26).

**Figure 11:**
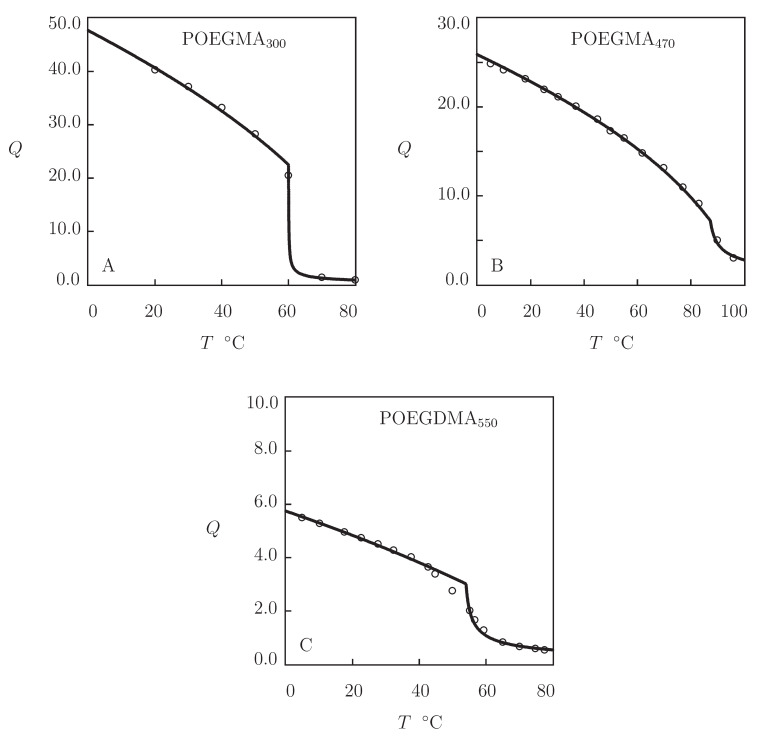
Degree of swelling *Q* versus temperature *T*. Circles: experimental data ((**A**) POEGMA${}_{300}$ gel [105], (**B**) POEGMA${}_{470}$ nanocomposite gel [104], and (**C**) POEGDMA${}_{550}$ gel [106]). Solid lines: results of simulation.

All swelling diagrams in Figure 11 are adequately described by the model with the material constants listed in Table S11. This table shows that POEGMA and POEGDMA gels with relatively high molar masses exhibit VPT, and their volume phase transition temperature $T_{c}$ increases with the molar mass (number of ethylene glycol (EG) units) of monomers.

**Figure 12:**
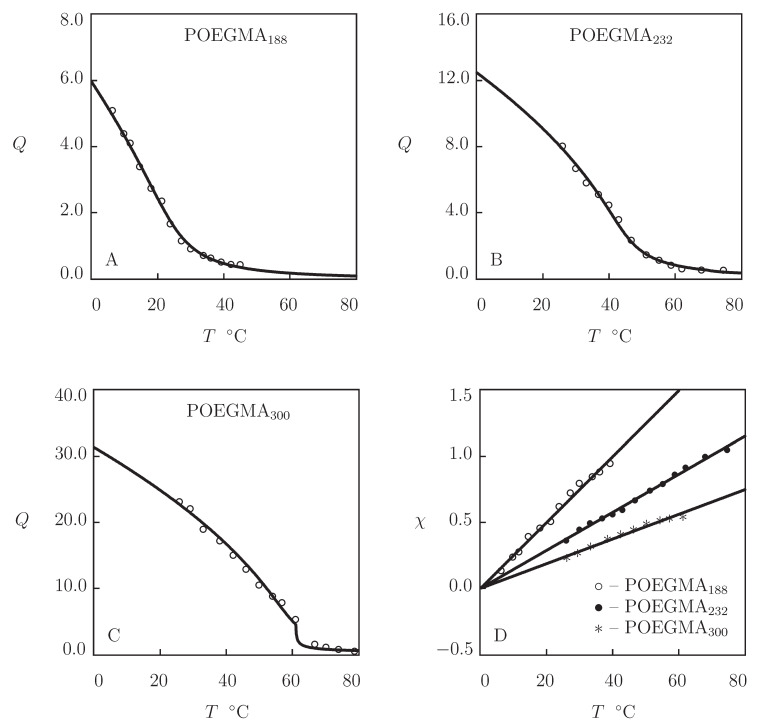
(**A**–**C**) Degree of swelling *Q* versus temperature *T*. Circles: experimental data [28] on POEGMA${}_{188}$, POEGMA${}_{232}$ and POEGMA${}_{300}$ microgels. Solid lines: results of simulation. (**D**) Parameter χ versus temperature *T*. Symbols: treatment of experimental data. Solid lines: results of simulation.

To demonstrate that POEGMA gels with small number of EG units (low hydrophilicity) do not show VPT, we analyze observations on a series of microgels prepared by polymerization of oligomers with various chain lengths. Observations in equalibrium water uptake tests on microgels prepared by polymerization of di(ethylene glycol) methyl ether methacrylate (molar mass 188 g/mol), tri(ethylene glycol) methyl ether methacrylate (molar mass 232 g/mol), and oligo(ethylene glycol) methyl ether methacrylate (molar mass 300 g/mol) are depicted in Figure 12. The gels were synthesized by free radical precipitation polymerization (4 h at 90 °C) of an aqueous solution of monomers (molar fraction 95 mM) by using EGDMA (molar fraction with respect to monomers 0.02) as a cross-linker, and KPS as an initiator (Tatry et al. [28]).

Experimental data in Figure 12 are approximated by the model with the material constants collected in Table S12. This table shows that POEGMA${}_{188}$ and POEGMA${}_{232}$ microgels do not exhibit VPT, while the POEGMA${}_{300}$ microgel demonstrates volume phase transition at the temperature $T_{c}$ close to that for the POEGMA${}_{300}$ gel in Figure 11A. To verify these results, the experimental dependencies of the FH parameter χ(T) are reported in Figure 12D together with their fits by Equation (26). This figure shows that the data for POEGMA${}_{188}$ and POEGMA${}_{232}$ are in good accord with the theoretical curves in the entire interval of temperatures under consideration, while those for POEGMA${}_{300}$ are adequately described by Equation (26) below VPTT only.

We proceed with matching observations on POEGMA macro- and microgels reported in Figure 11 and Figure 12.

Figure 11 presents experimental swelling diagrams on two POEGMA gels with molecular weights 300 and 470 g/mol and a poly(oligo(ethylene glycol) dimethacrylate) (POEGDMA) gel with molecular weight 550 g/mol. The data in Figure 11A were obtained in equilibrium swelling tests on POEGMA${}_{300}$ gel prepared by cross-linking polymerization (12 h at 70 °C) of a solution of OEGMA monomers in 2-propanol by using EGDMA (molar fraction with respect to monomers 0.03) as a cross-linker and AIBN as an initiator (Khodeir et al. [105]).

Observations in Figure 11B were obtained on physically cross-linked POEGMA${}_{470}$ gel prepared by free radical polymerization (24 h at room temperature) of an aqueous solution of OEGMA monomers (mass fraction 0.1) and Laponite XLS nanoclay (mass fraction 0.05) by using KPS as an initiator and TEMED as an accelerator (Xia et al. [104]). Figure 11C presents experimental data on POEGDMA${}_{550}$ gel prepared by γ-irradiation (the irradiation dose 25 kGy) of a solution of monomers (mass fraction 0.1) in a 1:1 (*v/v*) mixture of water and ethanol under ambient conditions (Suljovrujic et al. [106]).

**Figure 13:**
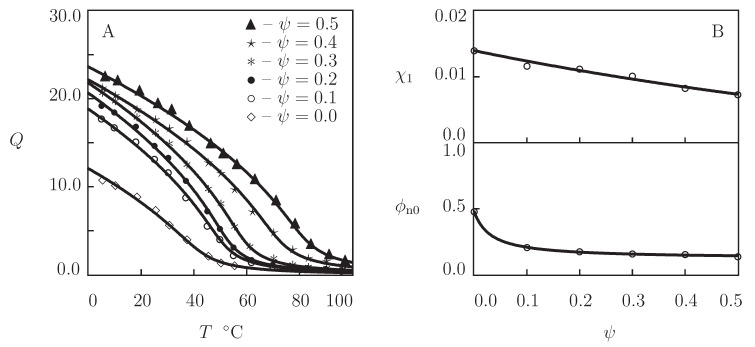
(**A**) Degree of swelling *Q* versus temperature *T*. Symbols: experimental data [104] on P(MEO${}_{2}$MA–OEGMA${}_{475}$) gels with various molar fractions ψ of OEGMA monomers. Solid lines: results of simulation. (**B**) Parameters $\chi_{1}$ and $\phi_{n0}$ versus molar fraction of OEGMA monomers ψ. Circles: treatment of observations. Solid lines: results of simulation.

To analyze equilibrium swelling of P(MEO${}_{2}$MA-OEGMA) copolymer gels with various molar fractions ψ of OEGMA monomers, we focus on three sets of experimental data. The first set of observations was obtained on nanocomposite gels prepared by copolymerization (24 h at room temperature) of an aqueous solution of MEO${}_{2}$MA and OEGMA${}_{475}$ monomers (mass fraction 0.1) and Laponite XLS nanoclay (mass fraction 0.05) by using KPS as an initiator and TEMED as an accelerator (Xia et al. [104]). Experimental data on copolymer gels with ψ=0.0, 0.1, 0.2, 0.3, 0.4, and 0.5 are presented in Figure 13A together with their fits by the model. Simulation is conducted with $g_{1}=0.017$ (the dimensionless elastic modulus found by matching observations on PMEO${}_{2}$MA homopolymer gel). Each equilibrium swelling curve in Figure 13A is characterized by two parameters: $\chi_{1}$ and $\phi_{n0}$. Evolution of these quantities with ψ is illustrated in Figure 13B, where the data are approximated by Equations (30) and (31).

Figure 13A shows that the P(MEO${}_{2}$MA-OEGMA${}_{475}$) copolymer gels do not suffer the volume phase transition. This conclusion is confirmed by the data presented in Figure S5. This figures reveals that for each ψ under consideration, evolution of the FH parameter χ with temperature *T* is correctly described by Equation (27) in the entire interval of temperatures between 0 and 100 °C.

**Figure 14:**
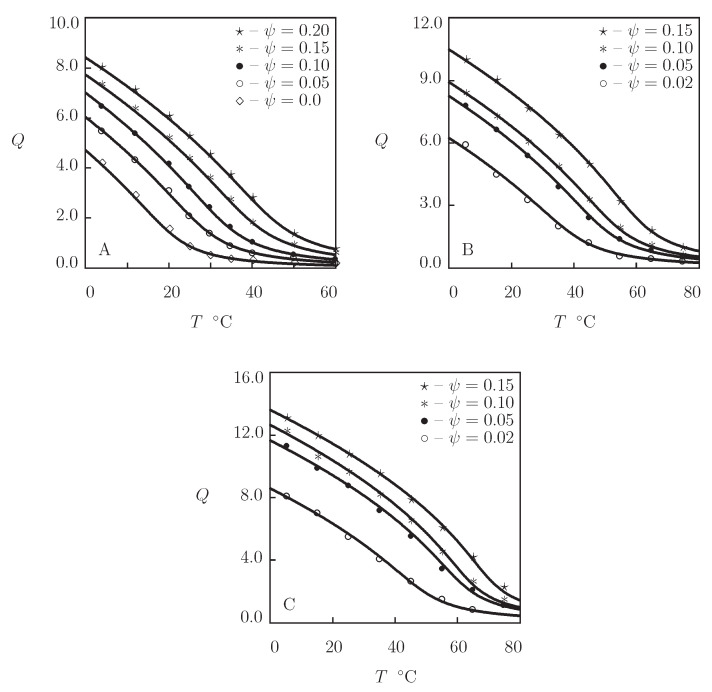
Degree of swelling *Q* versus temperature *T*. Symbols: experimental data [107] on P(MEO${}_{2}$MA-OEGMA${}_{M}$) copolymer gels with various molar fractions ψ and molar masses *M* of OEGMA monomers. (**A**) M=475, (**B**) M=1100, and (**C**) M=2080 g/mol. Solid lines: results of simulation.

The other set of observations is presented in Figure 14. Equilibrium water uptake tests were conducted on three series of P(MEO${}_{2}$MA-OEGMA) copolymer gels with various molar masses of OEGMA oligomers (M=475, 1100, and 2080 g/mol, which corresponded to 8, 23, and 45 EG units). The gels were prepared by cross-linking copolymerization (24 h at room temperature) of solutions of monomers (total concentration 1 g/mL) in water/ethanol mixtures by using tetraethylene glycol dimethacrylate (TEGDMA, mass fraction with respect to monomers 0.005) as a cross-linker, APS as an initiator and TEMED as an accelerator (Paris and Quijada-Garrido [107]).

The dimensionless elastic modulus $g_{1}=0.05$ is found by matching the experimental swelling diagram on PMEO${}_{2}$MA homopolymer gel (Figure 14A). Afterwards, each set of observations in Figure 14A–C is fitted by means of two coefficients, $\chi_{1}$ and $\phi_{n0}$, only. Evolution of these parameters with molar fraction ψ of OEGMA monomers is demonstrated in Figure S6, where the data are approximated by Equations (30) and (31).

To confirm that P(MEO${}_{2}$MA-OEGMA) copolymer gels do not exhibit VPT in the entire interval of temperatures under investigation, the FH parameter χ is plotted as a function of temperature *T* in Figure S7. For illustration, only data on P(MEO${}_{2}$MA-OEGMA${}_{475}$) and P(MEO${}_{2}$MA-OEGMA${}_{2080}$) are reported together with their approximations by Equation (27). This figure shows that the experimental dependencies χ(T) for copolymer gels with all molar masses of OEGMA oligomers are correctly described by Equation (27).

**Figure 15:**
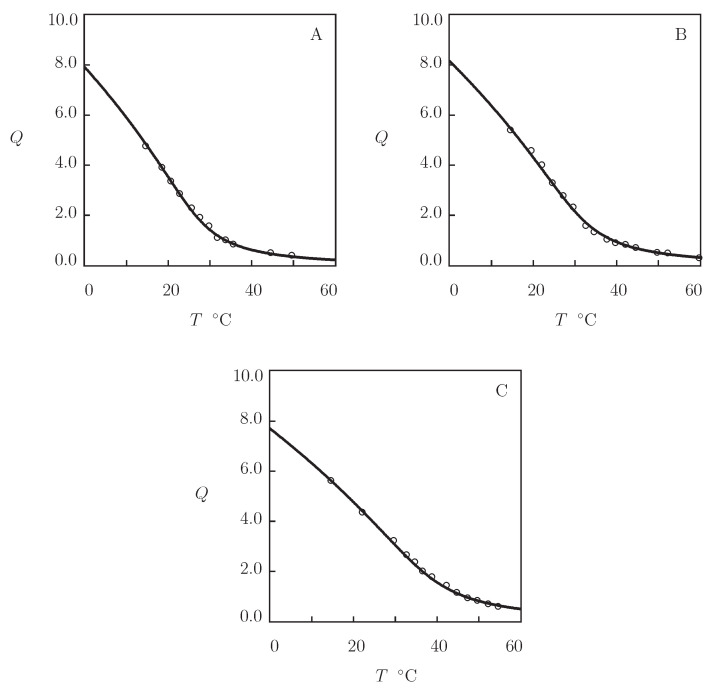
Degree of swelling *Q* versus temperature *T*. Circles: experimental data [108] on P(MEO${}_{2}$MA-OEGMA${}_{500}$) microgels with various molar fractions ψ of OEGMA monomers ((**A**) ψ=0.05, (**B**) ψ=0.17, and (**C**) ψ=0.26). Solid lines: results of simulation.

We now study equilibrium swelling curves (Figure 15) on P(MEO${}_{2}$MA-OEGMA${}_{500}$) copolymer microgels with molar fractions of OEGMA monomers ψ=0.05, 0.17, and 0.26. The microgels were synthesized by precipitation polymerization (4 h at 70 °C of aqueous solutions of comonomers by using EGDMA (molar fraction with respect to monomers 0.03) as a cross-linker, KPS as an initiator, and Glucopon 220 (molar fraction with respect to monomers 0.009) as a surfactant (Gawlitza et al. [108]).

Figure 15A–C demonstrates reasonable agreement between the experimental data and results of simulation with the only adjustable parameter $\chi_{1}$ (the coefficients $g_{1}=0.02$ and $Q_{0}=0.1$ are found by matching observations on the microgel with ψ=0.05 and used without changes to fit the data on the other gels). The coefficient $\chi_{1}$ is determined by approximation of the dependence of the FH parameter χ on temperature *T* (Figure S8A) by means of Equation (26). Figure S8A shows that the microgels do not reveal VPT within the interval of temperatures under consideration (each set of data is adequately described by Equation (26)). The coefficient $\chi_{1}$ decreases slightly with molar fraction ψ of OEGMA monomers (Figure S8B). Changes in $\chi_{1}$ with ψ are correctly predicted by Equation (30) with *A* close to unity.

To examine the response of copolymer gels with a similar chemical structure, we approximate experimental swelling diagrams on poly(2-methoxyethyl acrylate-co-(ethylene glycol) methyl ether acrylate) P(MEA-OEGA) microgels with two molar fractions ψ=0.1 and 0.3 of OEGA${}_{480}$ monomers (Figure S9). The gels were synthesized by means of the RAFT dispersion copolymerization (at 70 °C of a solution of MEA and OEMA monomers in $N,N^{\prime }$-dimethylformamide (DMF) by using benzyl ethyl trithiocarbonate as the chain transfer agent (CTA), poly(ethylene glycol) diacrylate (PEGDA, molar fraction 0.03) as a cross-linker, and 2,2${}^{\prime }$-azobis(2-methylpropionamidine) dihydrochioride (V-50) as an initiator (Liu et al. [109]). Figure S9A,B demonstrates good agreement between the observations and results of simulation with the material parameters reported in Table S13. This table shows that $\chi_{1}$ and $Q_{0}$ are practically unaffected by molar fraction of OEGA monomers, whereas $g_{1}$ decreases slightly with ψ. Figure S9C,D reveals that the microgels do not exhibit VPT (the growth of the FH parameter χ with temperature *T* is correctly described by Equation (26) in the entire interval of temperatures under consideration).

## 4. Discussion and Conclusions

This study deals with the analysis of equilibrium swelling of thermo-responsive homo- and copolymer gels to be employed in biomedical applications. Due to the concern regarding cytotoxicity of substituted acrylamide gels, we focus on the response of five groups of biocompatible hydrogels with poly(vinylcaprolactam), poly(vinyl methyl ether), poly(dimethylaminoethyl methacrylate), poly(oxazoline)s, and poly(methoxyethoxy ethyl methacrylate) as temperature-sensitive monomers. The aim is to compare their equilibrium water uptake curves with those on substituted acrylamide gels and to assess how molar fraction of comonomers affects VPTT of these gels.

For this purpose, a unified model is developed for equilibrium swelling of TR gels. The model is grounded on the conventional scenario, according to which the Flory–Huggins parameter χ of a TR gel increases with temperature below VPTT (due to thermally-induced breakage of cage-like structures formed by water molecules around hydrophobic segments) and reaches its ultimate value at the volume phase transition temperature. Transition of the gel from its swollen into collapsed state is driven by aggregation of hydrophobic segments into clusters that serve as extra physical bonds between polymer chains above VPTT. Material constants in the governing equations are found by matching experimental swelling diagrams. Good agreement is demonstrated between the observations and results of numerical simulation.

The entire set of biocompatible thermo-responsive gels under consideration can be split into three groups depending on the strength of hydrophobic interactions between segments.

TR gels with strong hydrophobic interactions (PNIPAm and PVCL) demonstrate abrupt volume phase transitions. Their homo- and copolymer gels expel practically all water molecules when they are in the collapsed state (Figure 1 and Figure 3).

TR gels with intermediate hydrophobic interactions (PMVE and PDMAEMA) show sharp volume phase transitions from the swollen state into a sponge-like state with degrees of swelling above VPTT ranging from 2 to 10 (Figure 5 and Figure 6). This feature is attributed to the fact that hydrophobic interactions with moderate strength cannot resist entirely elastic forces in the polymer network (PMVE) and repulsive forces between ionized functional groups (PDMAEMA) that impede formation of hydrophobic clusters.

The volume phase transition temperature of copolymer gels prepared by copolymerization of TR monomers with strong and intermediate hydrophobic interactions and temperature-insensitive monomers is adequately predicted by Equation (23), see Figure 2, Figure 4, and Figure 6.

TR gels with weak hydrophobic interactions (POx and PMEO${}_{2}$MA) do not exhibit volume phase transitions. Although their swelling diagrams demonstrate a pronounced decay in the equilibrium degree of swelling with temperature, these curves are correctly described by the model without assumption about formation of hydrophobic clusters. Two reasons are proposed to explain this behavior: (i) elasticity of the polymer network hinders aggregation of hydrophobic segments, and (ii) thermally-induced expulsion of water molecules from the gels makes the aggregation process thermodynamically unfavorable.

According to this classification, POEGMA gels are located on the border between TR gels with weak and intermediate strength of hydrophobic interactions. When the molar mass of OEGMA monomers is low, the response of these gels is similar to that of POx and PMEO${}_{2}$MA gels. Their swelling diagrams resemble those on PMVE gels when the molar mass becomes relatively large.

Equilibrium swelling of TR gels prepared by polymerization of weakly hydrophobic monomers with comonomers is affected by the chemical structure of comonomers. TR gels prepared by copolymerization of PEtOx monomers with strongly hydrophobic monomers (HEMA, HPA, and MMA) demonstrate sharp volume phase transitions. TR gels manufactured by copolymerization of MEO${}_{2}$MA and MEA monomers with OEGMA and EOGA monomers (with weak hydrophobic interactions) do not exhibit VPT in the entire interval of temperatures between 0 and 100 °C.

## Data Availability

The data presented in this study are available on request from the author.

## Supplementary Materials

Supplementary Information is available online at https://www.mdpi.com/article/10.3390/gels7020040/s1.
