# Supplementary material for: Equilibrium Swelling of Biocompatible Thermo-Responsive Copolymer Gels
Source: Gels. 2021 Apr 1;7(2):40. doi: 10.3390/gels7020040 (PMC8167660; doi:10.3390/gels7020040)
Supplement: Supplementary file 1 [file gels-07-00040-s001.pdf]

Supplementary material
Equilibrium swelling of biocompatible thermo-responsive copolymer gels

Aleksey D. Drozdov*
Department of Materials and Production
Aalborg University
Fibigerstraede 16, Aalborg 9220, Denmark

*E–mail: aleksey@m-tech.aau.dk

Figures

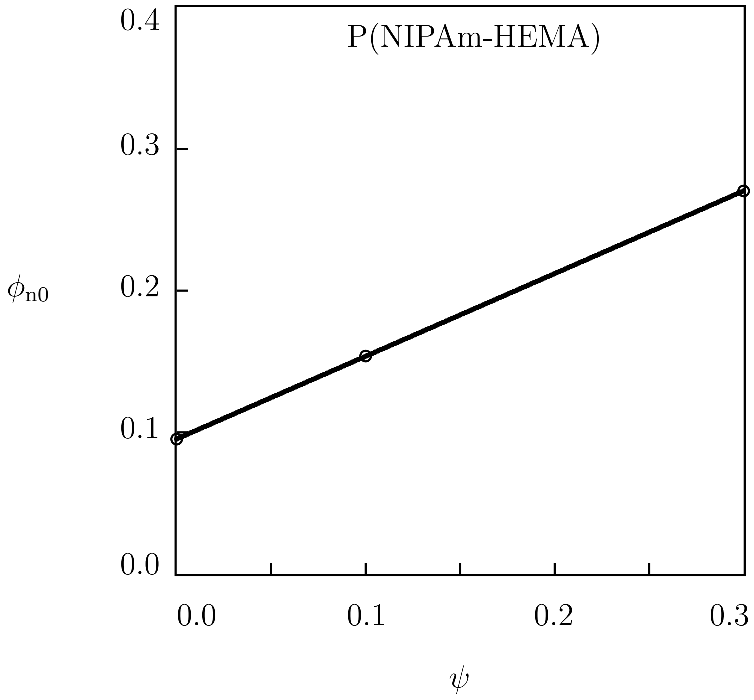

Figure S-1: Parameter ϕ_{n0} versus molar fraction of comonomers ψ. Circles: treatment of observations (Lee and Huang, 2000) on P(NIPAm-HEMA) gels. Solid line: results of simulation.

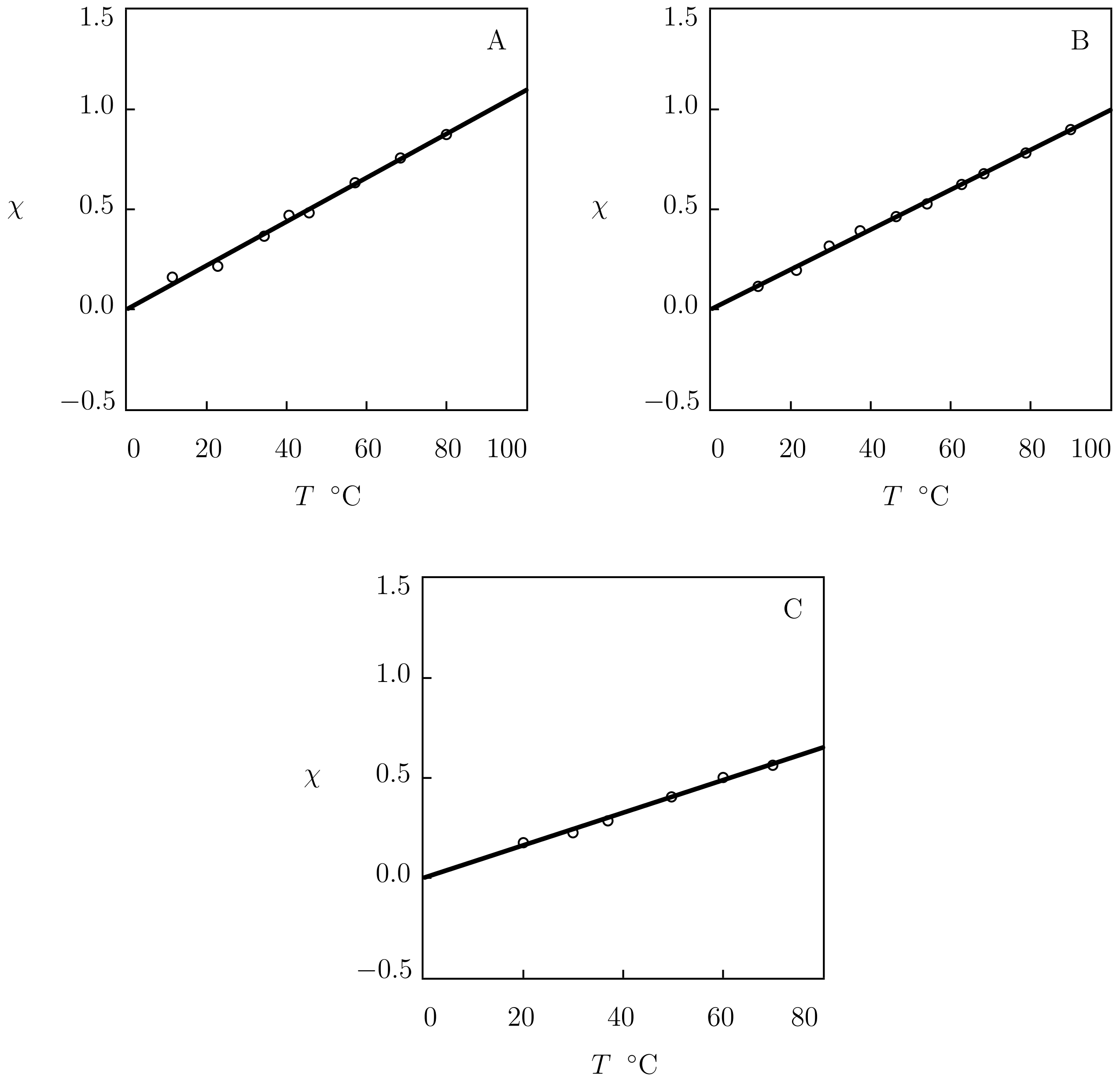

Figure S-2: Parameter χ versus temperature T. Circles: experimental data on POx gels. A – PEtOx, Christova et al. (2003), B – PEtOx, Seguet et al. (2020), C – PIPOx, Jerca et al. (2018). Solid lines: results of simulation.

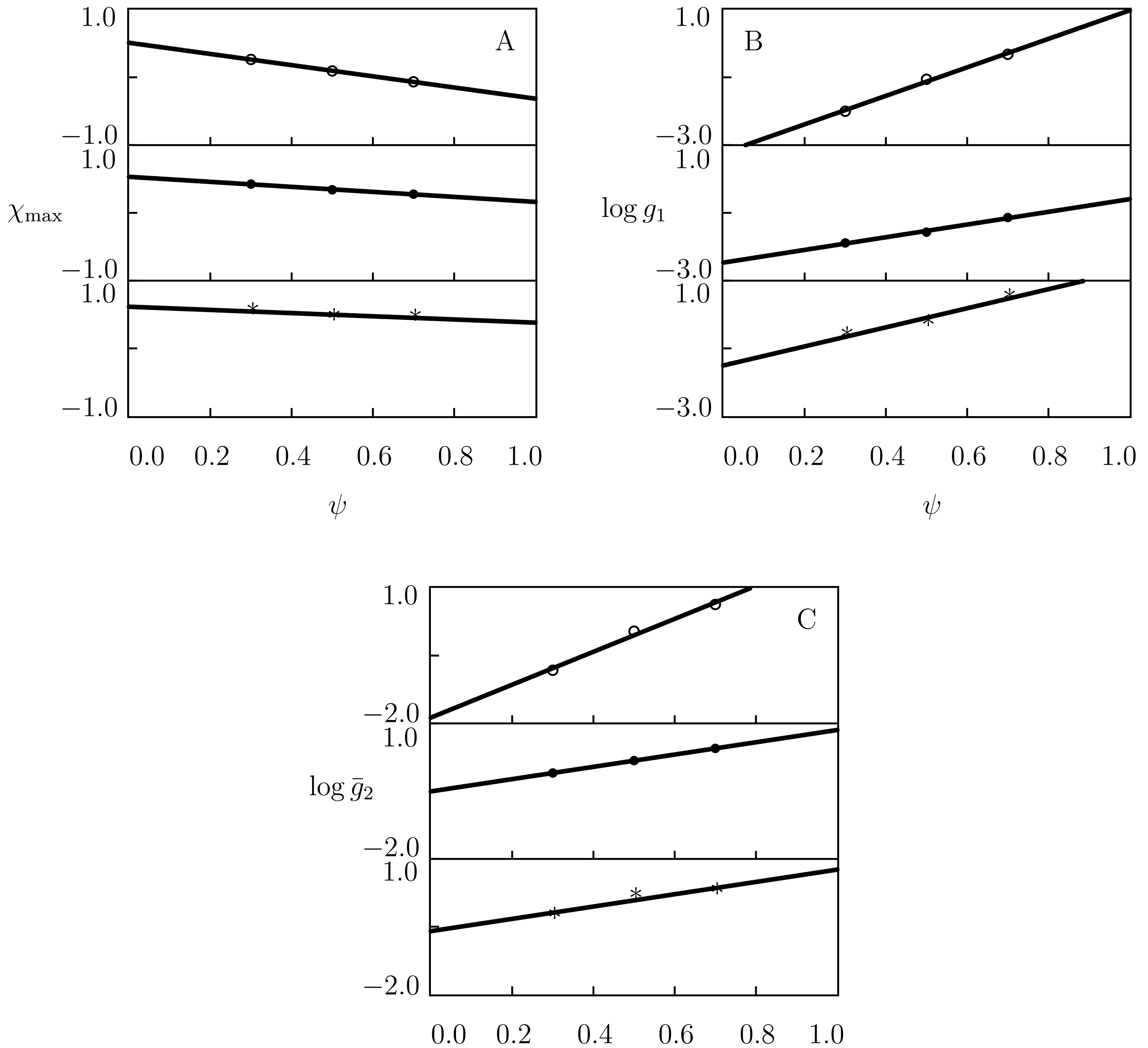

Figure S-3: Parameters χ_{max} (A), g_1 (B) and $\bar{g}_2$ (C) versus molar fraction ψ of comonomers. Symbols: treatment of observations on P(EtOx-HEMA), P(EtOx-HPA) and P(EtOx-MMA) copolymer gels ($\circ$ – HEMA, $\bullet$ – HPA, $*$ – MMA). Solid lines: results of simulation.

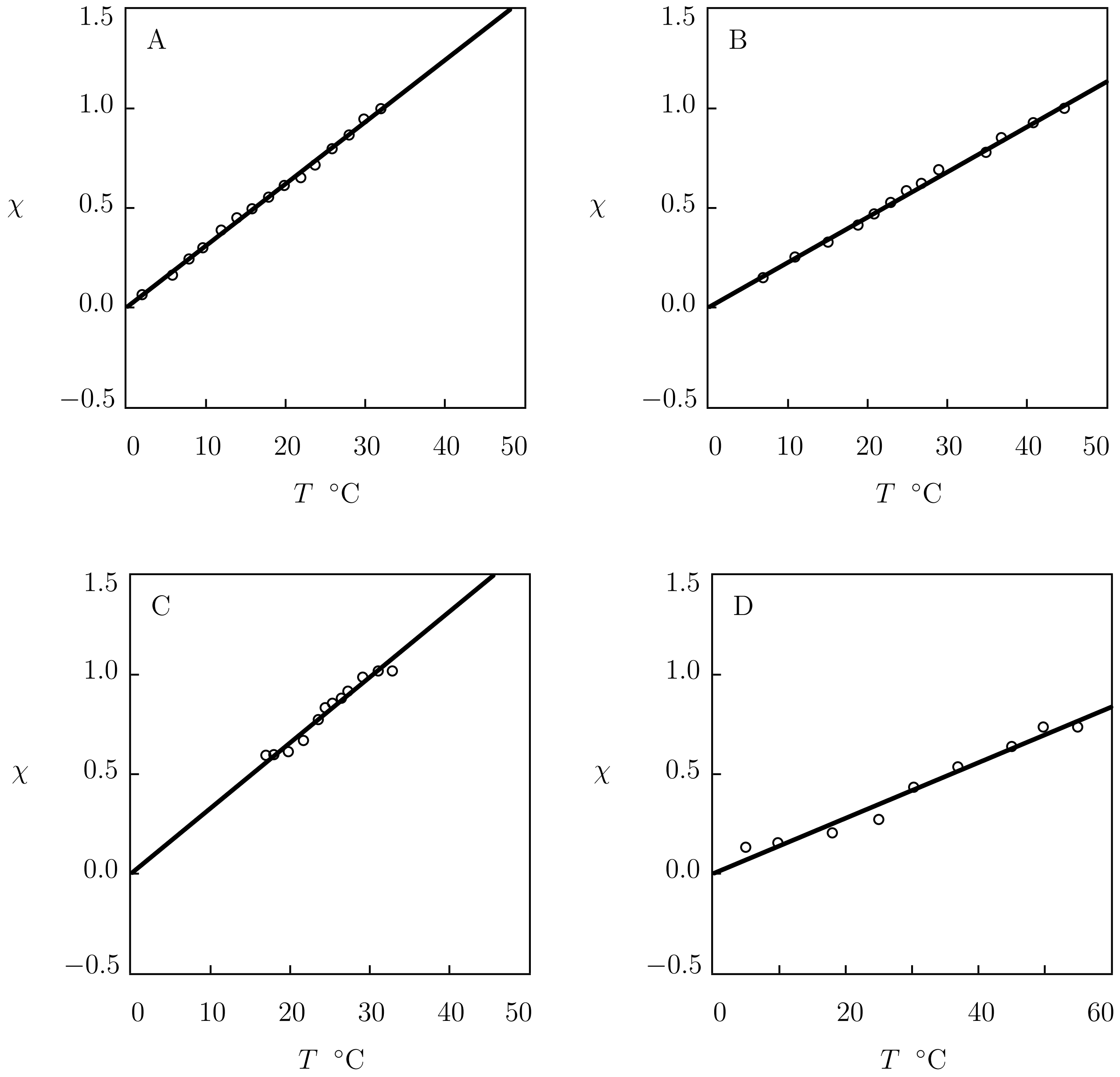

Figure S-4: Parameter χ versus temperature T. Circles: experimental data on $PMEO_2MA$ gels. Solid lines: results of simulation. A – macroscopic gel (Iizawa et al., 2012), B – core-shell microgel with Au core and gel shell (Lapresta-Fernandez et al., 2014), C – microgel particle (Cai et al., 2007), D – nanocomposite gel (Xia et al., 2015).

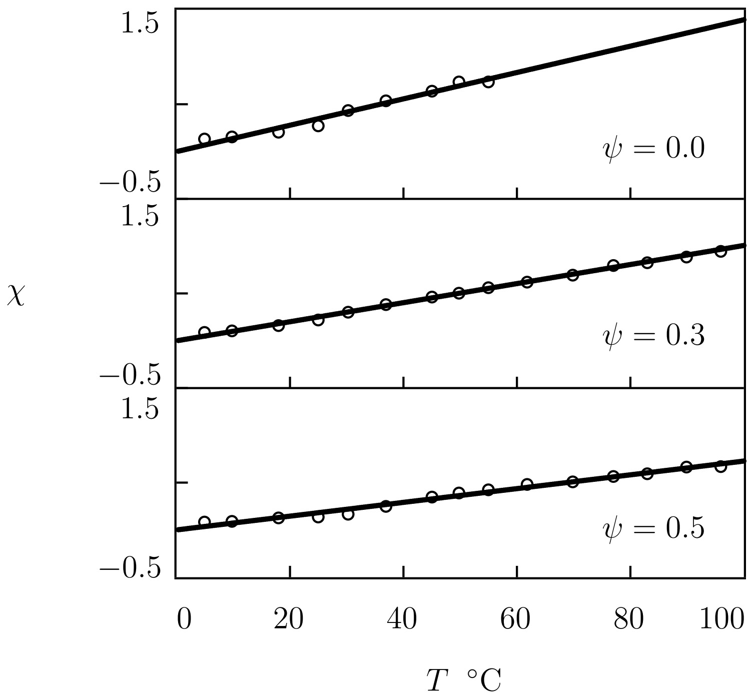

Figure S-5: Parameter χ versus temperature T. Circles: experimental data (Xia et al., 2015) on P(MEO_2MA–$OEGMA_{475}$) nanocomposite gels with various molar fractions ψ of OEGMA monomers. Solid lines: results of simulation.

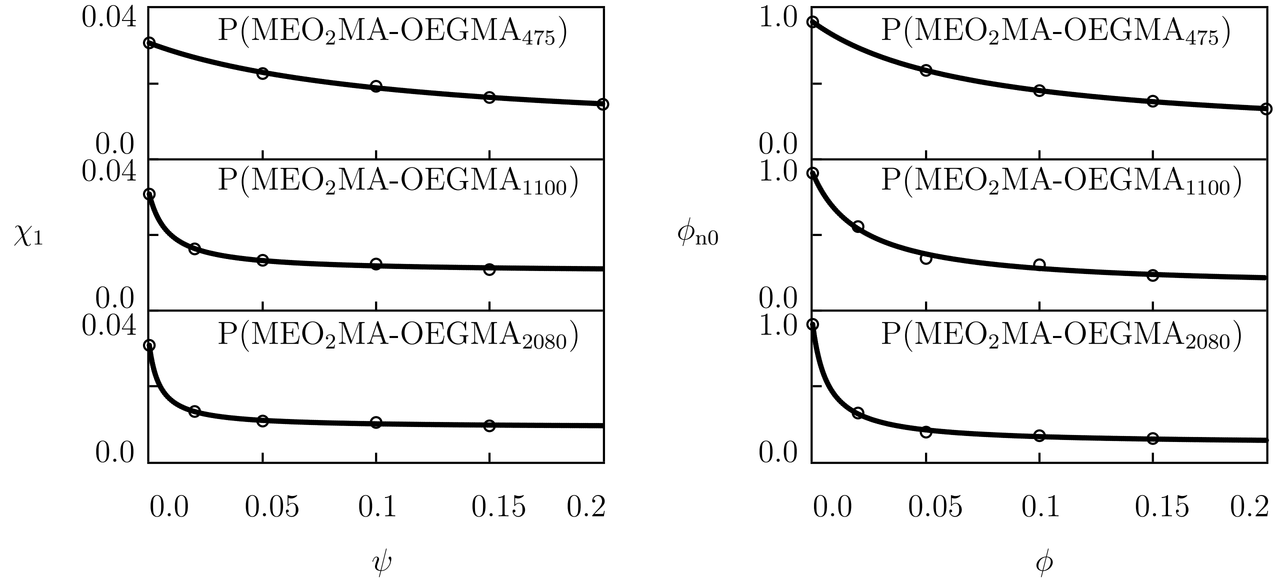

Figure S-6: Parameters χ_1 and $\phi_{\rm n0}$ versus molar fraction ψ of OEGMA monomers. Circles: treatment of observations on P(MEO_2MA-$OEGMA_M$) copolymer gels. Solid lines: results of simulation.

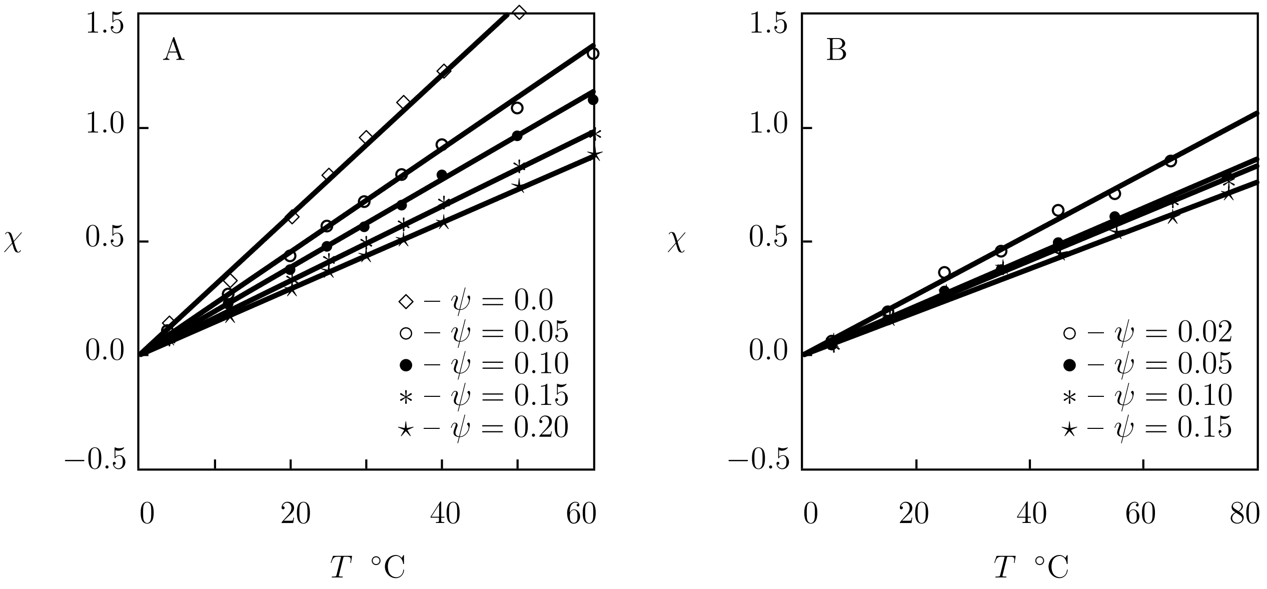

Figure S-7: Parameter χ versus temperature T. Symbols: experimental data (Paris and Quijada-Garrido, 2009) on P(MEO_2MA-$OEGMA_{475}$) (A) and P(MEO_2MA-$OEGMA_{2080}$) (B) copolymer gels with various molar fractions ψ of OEGMA monomers. Solid lines: results of simulation.

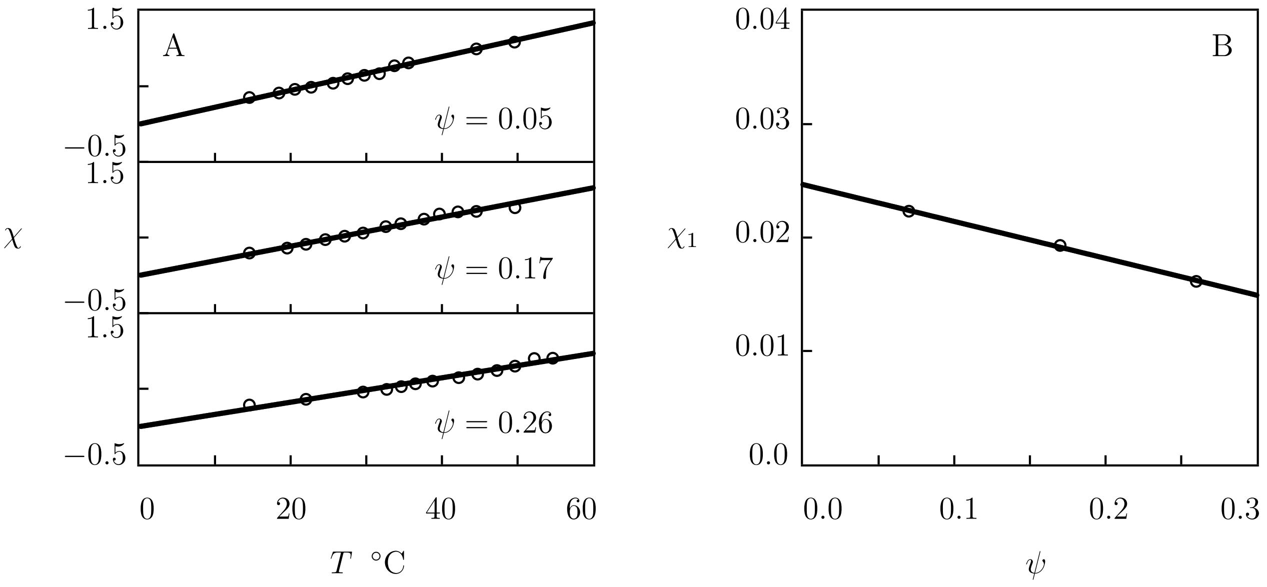

Figure S-8: A – Parameter χ versus temperature T. Circles: experimental data (Gawlitza et al., 2014) on P(MEO_2MA-$OEGMA_{500}$) microgels with various molar fractions ψ of OEGMA monomers. Solid lines: results of simulation. B – Parameter χ_1 versus molar fraction ψ of OEGMA monomers. Circles: treatment of observations. Solid line: results of simulation.

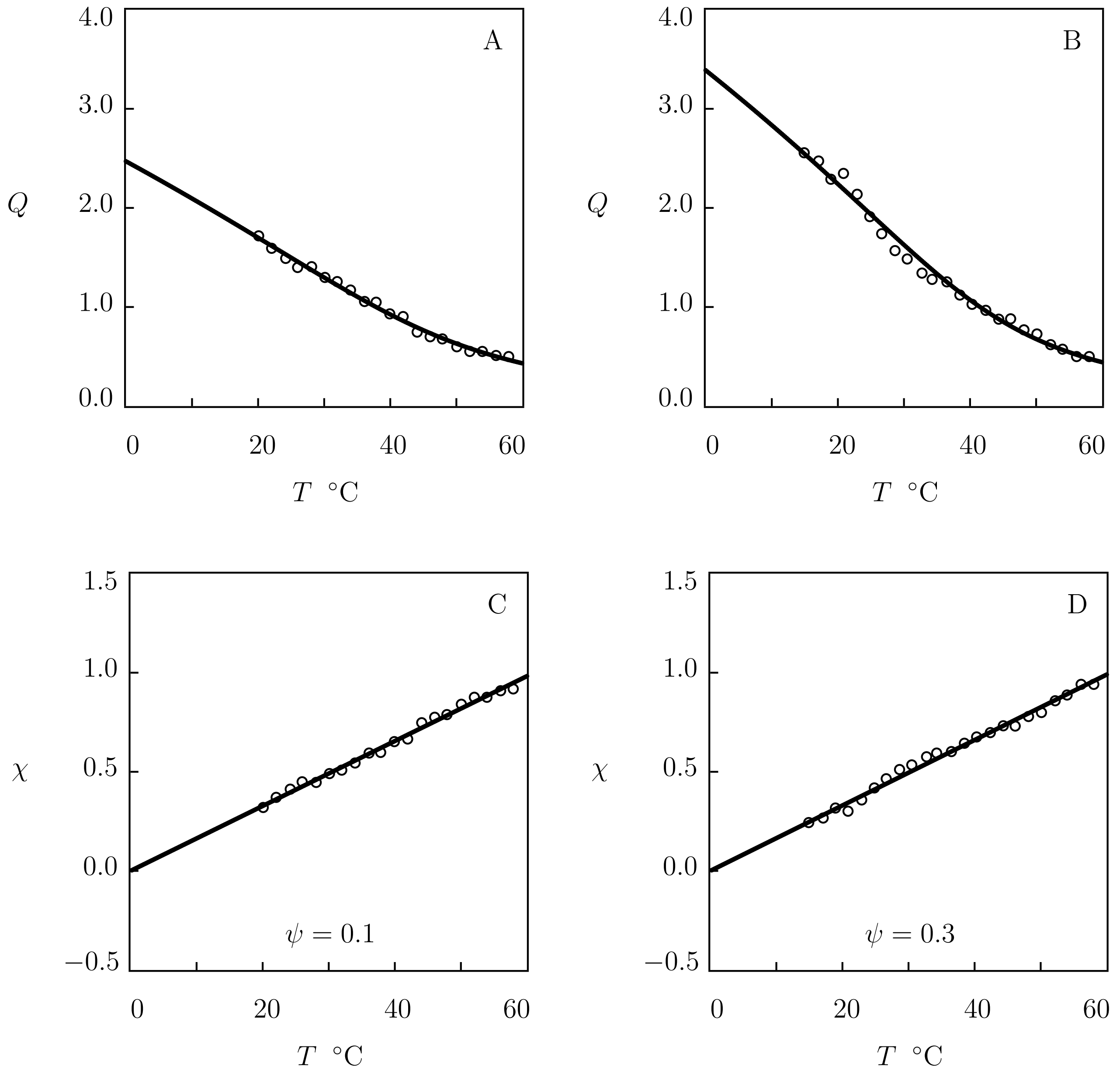

Figure S-9: A, B – Degree of swelling Q versus temperature T. Circles: experimental data (Liu et al., 2013) on (MEA-OEGA) microgels with molar fractions $\psi = 0.1$ (A) and 0.3 (B) of $OEGA_{480}$ monomers. Solid lines: results of simulation. C, D – Parameter χ versus temperature T. Circles: treatment of experimental data. Solid lines: results of simulation.

Tables

Table S-1: Material parameters for N-substituted acrylamide gels.

Gel	χ_0	χ_1	$\chi_{\max}$	g_1	Q_0	$\bar{g}_2$	β	T_c °C
PNNPAm	-3.147	0.123	-0.10	0.075	13.3	2.00	0.60	24.8
PNIPAm	-2.098	0.079	0.60	0.075	13.3	0.45	0.45	34.1
PNCPAm	-0.752	0.031	0.70	0.075	13.3	0.45	0.15	46.1
PDEAm	0.167	0.018	0.82	0.050	4.0	3.00	0.50	35.9

Table S-2: Material parameters for P(NIPAm-HEMA) gels with various molar fractions ψ of HEMA monomers.

ψ	χ_0	χ_1	$\chi_{\max}$	g_1	Q_0	$\bar{g}_2$	β	T_c °C
0.0	-2.098	0.0791	0.49	0.123	9.5	0.8	0.4	32.7
0.1	-1.732	0.0712	0.49	0.123	5.5	0.8	0.4	31.2
0.3	-0.999	0.0554	0.49	0.123	2.7	0.8	0.4	26.9

Table S-3: Material parameters for poly(N-vinylcaprolactam) macro- and microgels.

Figure	χ_0	χ_1	$\chi_{\max}$	g_1	$\bar{g}_2$	β	β_1	T_c °C
4A	-0.463	0.0396	0.72	0.05	0.18	0.04	1.5	29.9
4B	-0.463	0.0396	0.86	0.23	2.50	0.40	1.5	33.5
4C	-0.463	0.0396	0.71	0.20	0.80	0.05	2.0	29.7
4D-BIS	-0.463	0.0396	0.69	0.07	2.00	0.01	0.6	29.2
4D-PEGDA	-0.463	0.0396	0.69	0.10	2.50	0.004	0.3	29.2

Table S-4: Material parameters for P(VCL-MEA) microgels with various molar fractions ψ of MEA monomers.

ψ	χ_0	χ_1	$\chi_{\max}$	g_1	Q_0	$\bar{g}_2$	β	β_1	T_c °C
0.035	-0.409	0.0382	0.71	0.096	10.3	1.5	0.04	2.5	29.3
0.30	-0.301	0.0277	0.71	0.050	4.6	1.5	0.25	3.0	25.8

Table S-5: Material parameters for poly(vinyl methyl ether) gels.

Figure	χ_0	χ_1	$\chi_{\max}$	g_1	Q_0	$\bar{g}_2$	β	T_c °C
5A	0.384	0.0117	0.775	0.080	12.3	0.01	25.0	33.5
5B	0.384	0.0117	0.788	0.080	11.2	0.12	30.0	33.9
5C	0.384	0.0117	0.758	0.081	7.2	0.10	25.0	32.0
5D	0.384	0.0117	0.780	0.147	5.1	0.25	16.0	33.9

Table S-6: Material parameters for poly(N,N-dimethylaminoethyl methacrylate) gels.

Figure	χ_0	χ_1	$\chi_{\max}$	g_1	Q_0	$\bar{g}_2$	β	T_c °C
6A	-0.3	0.0136	0.310	0.006	0.1	0.400	8.0	44.9
6B	-0.3	0.0136	0.294	0.006	7.8	0.009	10.0	43.7
6C	-0.3	0.0136	0.294	0.005	8.8	0.009	10.0	43.7
6D	-0.3	0.0136	0.304	0.007	9.2	0.006	12.0	44.4

Table S-7: Material parameters for P(DMAEMA-EAAm) and P(DMAEMA-AAm) gels with various molar fractions ψ of comonomers.

Comonomer	ψ	χ_0	χ_1	$\chi_{\max}$	g_1	Q_0	$\bar{g}_2$	β	T_c °C
EAAm									
	0.05	-0.410	0.0129	0.304	0.0067	9.9	0.004	12.0	55.3
	0.10	-0.535	0.0129	0.304	0.0067	7.6	0.004	12.0	65.0
AAm									
	0.20	-0.095	0.0109	0.304	0.0067	7.8	0.008	12.0	36.7
	0.33	0.002	0.0091	0.304	0.0067	8.5	0.008	12.0	31.2

Table S-8: Material parameters for poly(2-ethyl-2-oxazoline) and poly(2-isopropenyl-2-oxazoline) gels.

Figure	χ_1	g_1	Q_0
8A	0.0011	0.0310	7.5
8B	0.0099	0.0972	1.8
8C	0.0082	0.0170	0.3

Table S-9: Material parameters for P(EtOx-HEMA), P(EtOx-HPA) and P(EtOx-MMA) gels with various molar fractions ψ of comonomers.

Comonomer	ψ	χ_0	χ_1	$\chi_{\max}$	g_1	Q_0	$\bar{g}_2$	β	T_c °C
HEMA									
	0.3	-0.090	0.0077	0.265	0.010	0.1	0.15	4.5	46.2
	0.5	-0.015	0.0055	0.095	0.088	0.1	1.10	4.5	44.7
	0.7	-0.210	0.0033	-0.065	0.481	0.1	4.30	4.5	44.1
HPA									
	0.3	0.057	0.0077	0.425	0.013	0.1	0.80	4.5	47.9
	0.5	0.095	0.0055	0.342	0.027	0.1	1.50	4.5	45.0
	0.7	0.133	0.0033	0.277	0.073	0.1	2.80	4.5	43.8
MMA									
	0.3	0.135	0.0077	0.559	0.255	0.1	0.57	4.5	55.2
	0.5	0.175	0.0055	0.470	0.591	0.1	1.55	4.5	53.8
	0.7	0.315	0.0033	0.465	3.408	0.1	2.00	4.5	45.6

Table S-10: Material parameters for $PMEO_2MA$ macro- and microgels.

Figure	χ_1	g_1	Q_0
10A	0.0316	0.073	1.3
10B	0.0227	0.032	0.1
10C	0.0329	0.048	6.2
10D	0.0139	0.017	1.1

Table S-11: Material parameters for POEGMA and POEGDMA gels.

Gel	χ_1	χ_{max}	g_1	Q_0	$\bar{g}_2$	β	T_{c} °C
POEGMA_{300}	0.0067	0.41	0.004	6.3	14.0	0.22	60.0
POEGMA_{470}	0.0063	0.55	0.017	7.7	4.0	0.05	87.3
POEGDMA_{550}	0.0083	0.45	0.070	1.1	19.0	0.25	54.1

Table S-12: Material parameters for POEGMA microgels.

Gel	χ_1	g_1	χ_{max}	Q_0	$\bar{g}_2$	β	T_{c} °C
POEGMA_{188}	0.0249	0.033		0.1			
POEGMA_{232}	0.0144	0.043		4.1			
POEGMA_{300}	0.0093	0.007	0.57	4.5	10.0	0.4	61.1

Table S-13: Material parameters for (MEA-OEGA) microgels with various molar fractions ψ of OEGA monomers.

ψ	χ_1	g_1	Q_0
0.1	0.0164	0.156	0.1
0.3	0.0165	0.089	0.1
